# Supplementary material for: Development and validation of a prognostic tool: Pulmonary embolism short-term clinical outcomes risk estimation (PE-SCORE)
Source: PLoS One. 2021 Nov 18;16(11):e0260036. doi: 10.1371/journal.pone.0260036 (PMC8601564; doi:10.1371/journal.pone.0260036)
Supplement: S1 Table — (DOCX) [file pone.0260036.s001.docx]

**Supplemental Table 1: Univariable analysis of 138 candidate variables for primary outcome on development database**

|  | Clinical Deterioration at 5 Days | No Clinical Deterioration  at 5 Days | P-value* |
| --- | --- | --- | --- |
|  | (N = 209) | (N = 726) |  |
| Age |  |  |  |
| Mean (SD) | 62.4 (16.5) | 59.7 (16.4) | 0.042 |
| Median [Min, Max] | 66.0 [20.0, 93.0] | 61.5 [18.0, 104] |  |
| Age > 80 years    Systolic BP, mmHg | 20 (15.3%) | 33 (11.4%) | 0.06 |
| Mean (SD) | 121 (25.9) | 135 (23.6) | <0.001 |
| Median [Min, Max] | 120 [55.0, 205] | 135 [69.0, 223] |  |
| Systolic BP < 100 mmHg    Initial Heart Rate (beats/min) | 29 (22.1%) | 23 (7.9%) | <0.01 |
| Mean (SD) | 107 (24.0) | 96.4 (20.2) | <0.001 |
| Median [Min, Max] | 109 [35.0, 184] | 96.0 [46.0, 181] |  |
| Missing | 1.00 (0.5%) | 0 (0%) |  |
| Abnormal heart rate  < 50 or > 100 beats/min    Shock Index Calculation | 89 (67.9%) | 134 (46.2%) | <0.01 |
| Mean (SD) | 0.920 (0.289) | 0.735 (0.218) | <0.001 |
| Median [Min, Max] | 0.900 [0.300, 2.00] | 0.700 [0.300, 1.70] |  |
| Missing | 1.00 (0.5%) | 0 (0%) |  |
| Initial Respiratory Rate |  |  |  |
| Mean (SD) | 21.2 (5.71) | 19.7 (4.25) | <0.001 |
| Median [Min, Max] | 20.0 [12.0, 39.0] | 18.0 [10.0, 48.0] |  |
| Missing | 1.00 (0.5%) | 3.00 (0.4%) |  |
| Initial Pulse Oximetry (%) |  |  |  |
| Mean (SD) | 93.8 (6.09) | 95.9 (4.37) | <0.001 |
| Median [Min, Max] | 95.0 [58.0, 100] | 97.0 [37.0, 100] |  |
| Missing | 2.00 (1.0%) | 1.00 (0.1%) |  |
| Initial Temperature (F) |  |  |  |
| Mean (SD) | 97.8 (1.35) | 98.2 (0.821) | <0.001 |
| Median [Min, Max] | 97.9 [89.0, 103] | 98.1 [94.2, 103] |  |
| Missing | 8.00 (3.8%) | 12.0 (1.7%) |  |
| Body Mass Index (Kg/m^2^) |  |  |  |
| Mean (SD) | 31.0 (8.50) | 31.3 (8.73) | 0.662 |
| Median [Min, Max] | 29.6 [16.1, 60.6] | 29.9 [14.1, 79.9] |  |
| Missing | 5.00 (2.4%) | 13.0 (1.8%) |  |
| Point-of-care BNP Level, pg/ml |  |  |  |
| Mean (SD) | 538 (828) | 191 (371) | <0.001 |
| Median [Min, Max] | 169 [5.00, 4670] | 58.5 [4.00, 4010] |  |
| Missing | 116 (55.5%) | 218 (30.0%) |  |
| NT Pro BNP |  |  |  |
| Mean (SD) | 2670 (8460) | 1160 (3740) | 0.082 |
| Median [Min, Max] | 334 [10.0, 70000] | 104 [5.00, 32200] |  |
| Missing | 102 (48.8%) | 542 (74.7%) |  |
| Point-of-care Troponin ng/L |  |  |  |
| Mean (SD) | 3.42 (45.3) | 0.0857 (0.212) | 0.292 |
| Median [Min, Max] | 0.0500 [0, 648] | 0.0300 [0, 2.76] |  |
| Missing | 4.00 (1.9%) | 48.0 (6.6%) |  |
| Length of Stay, hours |  |  |  |
| Mean (SD) | 197 (220) | 91.0 (115) | <0.001 |
| Median [Min, Max] | 131 [2.20, 1570] | 55.3 [0, 1010] |  |
| Missing | 35.0 (16.7%) | 5.00 (0.7%) |  |
| Clinical Research Site |  |  |  |
| Carolinas Medical Center | 49.0 (23.4%) | 263 (36.2%) | <0.001 |
| San Diego | 16.0 (7.7%) | 173 (23.8%) |  |
| Vanderbilt University  Med Center | 33.0 (15.8%) | 101 (13.9%) |  |
| Utah University | 33.0 (15.8%) | 45.0 (6.2%) |  |
| Orlando Regional  Med Center | 30.0 (14.4%) | 75.0 (10.3%) |  |
| Christiana Care | 48.0 (23.0%) | 69.0 (9.5%) |  |
| Gender |  |  |  |
| Female | 104 (49.8%) | 351 (48.3%) | 0.778 |
| Male | 105 (50.2%) | 375 (51.7%) |  |
| Race |  |  |  |
| Black | 50.0 (23.9%) | 203 (28.0%) | 0.661 |
| White | 147 (70.3%) | 491 (67.6%) |  |
| American Indian /Alaskan Native | 2.00 (1.0%) | 5.00 (0.7%) |  |
| Asian | 2.00 (1.0%) | 8.00 (1.1%) |  |
| Pacific Islander/Native Hawaiian | 0 (0%) | 2.00 (0.3%) |  |
| Unknown/Other | 8.00 (3.8%) | 17.0 (2.3%) |  |
| Ethnicity |  |  |  |
| Hispanic or Latino | 12.0 (5.7%) | 61.0 (8.4%) | 0.195 |
| Not Hispanic or Latino | 191 (91.4%) | 634 (87.3%) |  |
| Unknown | 5.00 (2.4%) | 31.0 (4.3%) |  |
| Missing | 1.00 (0.5%) | 0 (0%) |  |
| Preceding Episode Syncope |  |  |  |
| Yes | 38.0 (18.2%) | 54.0 (7.4%) | <0.001 |
| Transient Hypotension  prior to Enrollment | |  |  |
| Yes | 37.0 (17.7%) | 31.0 (4.3%) | <0.001 |
| Preceding Bradycardia |  |  |  |
| Yes | 8.00 (3.8%) | 8.00 (1.1%) | 0.018 |
| Preceding Pulselessness |  |  |  |
| Yes | 10.0 (4.8%) | 2.00 (0.3%) | <0.001 |
| Prior diagnosis of PE or DVT |  |  |  |
| Yes | 57.0 (27.3%) | 173 (23.8%) | 0.354 |
| Family History of VTE |  |  |  |
| Yes | 13.0 (6.2%) | 46.0 (6.3%) | 1 |
| Creatinine > 2.0 mg/dL |  |  |  |
| Yes | 29.0 (13.9%) | 52.0 (7.2%) | 0.004 |
| Moderate or severe  liver disease |  |  |  |
| Yes | 5.00 (2.4%) | 15.0 (2.1%) | 0.987 |
| Clotting Disorders |  |  |  |
| Yes | 13.0 (6.2%) | 14.0 (1.9%) | 0.002 |
| Recent Hospitalization |  |  |  |
| Yes | 70.0 (33.5%) | 216 (29.8%) | 0.343 |
| Recent Trauma |  |  |  |
| Yes | 19.0 (9.1%) | 47.0 (6.5%) | 0.251 |
| Chronic Obstructive  Pulmonary Disease | |  |  |
| Yes | 41.0 (19.6%) | 95.0 (13.1%) | 0.025 |
| Any cancer |  |  |  |
| Yes | 52.0 (24.9%) | 178 (24.5%) | 0.987 |
| Heart Failure |  |  |  |
| Yes | 16.0 (7.7%) | 39.0 (5.4%) | 0.285 |
| Hypovolemia |  |  |  |
| Yes | 25.0 (12.0%) | 19.0 (2.6%) | <0.001 |
| Medical or Social reason for  treatment in Hospital >24 hours | |  |  |
| Yes | 151 (72.2%) | 375 (51.7%) | <0.001 |
| Missing | 3.0 (1.4%) | 2.0 (0.3%) |  |
| Natriuretic peptide elevation |  |  |  |
| Yes | 113 (54.1%) | 236 (32.5%) | <0.001 |
| Missing | 8.00 (3.8%) | 34.0 (4.7%) |  |
| Troponin Elevation |  |  |  |
| Yes | 98.0 (46.9%) | 171 (23.6%) | <0.001 |
| Missing | 1.00 (0.5%) | 11.0 (1.5%) |  |
| CT RV:LV Ratio 1.0 or more |  |  |  |
| Yes | 110 (52.6%) | 199 (27.4%) | <0.001 |
| Missing | 5.00 (2.4%) | 12.0 (1.7%) |  |
| Most Proximal Location  thrombus on CTPA    Saddle    Proximal pulmonary artery  Lobar  Segmental  Subsegmental    **GDE Score** | 27 (20.6%)  49 (37.4%)  324 (24.4%)  15 (11.5%)  846 (6.1%) | 54 (18.6%)  69 (23.8%)  95 (32.8%)  60 (20.7%)  12 (4.1%) | <0.01 |
| 0 | 84.0 (40.2%) | 520 (71.6%) | <0.001 |
| 1 | 27.0 (12.9%) | 45.0 (6.2%) |  |
| 2 | 39.0 (18.7%) | 83.0 (11.4%) |  |
| 3 | 53.0 (25.4%) | 63.0 (8.7%) |  |
| Missing | 6.00 (2.9%) | 15.0 (2.1%) |  |
| Poor LV Function |  |  |  |
| Yes | 22.0 (10.5%) | 46.0 (6.3%) | 0.048 |
| Missing | 7.00 (3.3%) | 12.0 (1.7%) |  |
| GDE Showing abnlRV |  |  |  |
| Yes | 119 (56.9%) | 191 (26.3%) | <0.001 |
| Missing** | 6.00 (2.9%) | 15.0 (2.1%) |  |
| If GDE >0, is it acute?  Yes  No  Indeterminate    Low-risk sPESI | 102 (77.9%)  15 (11.5%)  14 (10.7%) | 162(55.9%)  102(35.2%)  26 (9.0%) | <0.01 |
| Yes | 31.0 (14.8%) | 283 (39.0%) | <0.001 |
| Low-risk ESC |  |  |  |
| Yes | 1.00 (0.5%) | 76.0 (10.5%) | <0.001 |
| Major Bleeding within 5 days |  |  |  |
| Yes | 13.0 (6.2%) | 10.0 (1.4%) | <0.001 |
| Recurrence of VTE  within 30 days |  |  |  |
| No | 200 (95.7%) | 710 (97.8%) | 0.049 |
| Yes | 6.00 (2.9%) | 6.00 (0.8%) |  |
| Missing | 3.00 (1.4%) | 10.0 (1.4%) |  |
| Major Bleeding  within 30 days |  |  |  |
| No | 190 (90.9%) | 703 (96.8%) | <0.001 |
| Yes | 16.0 (7.7%) | 14.0 (1.9%) |  |
| Missing | 3.00 (1.4%) | 9.00 (1.2%) |  |
| Death within 30 days |  |  |  |
| No | 165 (78.9%) | 699 (96.3%) | <0.001 |
| Yes | 44.0 (21.1%) | 24.0 (3.3%) |  |
| Missing | 0 (0%) | 3.00 (0.4%) |  |
| * Continuous variables compared via two-sample t-test assuming unequal variance. Categorical variables tested via chi-square test.  ** The percentages of patients experiencing the primary outcome among those with GDE showing abnormal RV response as negative, positive, and missing were 14.4%, 38.4%, and 28.6%, respectively.  **Abbreviations**: BNP = brain natriuretic peptide; PE = pulmonary embolism; DVT = deep vein thrombosis; VTE = venous thromboembolism; ESC= European Society of Cardiology Pulmonary Embolism Management guidelines (2019)[^15^](https://paperpile.com/c/bwE7vJ/PyUn); CT = computed tomography; LV = left ventricle; RV = right ventricle; GDE = goal-directed echocardiography; sPESI = simplified Pulmonary Embolism Severity Index | | | |
|  |  |  |  |
