# Supplementary material for: Development and validation of a prognostic tool: Pulmonary embolism short-term clinical outcomes risk estimation (PE-SCORE)
Source: PLoS One. 2021 Nov 18;16(11):e0260036. doi: 10.1371/journal.pone.0260036 (PMC8601564; doi:10.1371/journal.pone.0260036)
Supplement: S2 Table — (DOCX) [file pone.0260036.s002.docx]

| **Supplemental Table 2: Comparison of clinical research sites in development database** | | | | | | | |
| --- | --- | --- | --- | --- | --- | --- | --- |
| **Mean value at individual sites** | **Site 1** | **Site 2** | **Site 3** | **Site 4** | **Site 5** | **Site 6** | **P-value** |
|  | CMC | SD | VUM | UU | ORM | CC |  |
| Patients enrolled in both databases, number | 312 | 189 | 134 | 78 | 105 | 117 |  |
| Age, years (mean) | 58.1 | 64.4 | 59.1 | 60.0 | 60.5 | 61.2 | 0.0025 |
| Charlson comorbidity index (median) | 1.0 | 1.0 | 1.0 | 1.0 | 1.0 | 1.0 | .091 |
| **Proportion of patients at clinical sites with following components, %** | | | | | | | |
| Systolic blood pressure <100 mmHg, % | 9.9 | 4.2 | 11.2 | 7.7 | 6.7 | 12.8 | .092 |
| Abnormal Heart rate (<50 or >100 beats/min, % | 45.2 | 33.9 | 57.9 | 42.3 | 79.5 | 58.1 | <.0001 |
| Creatinine > 2.0 mg/dL | 5.1 | 1.1 | 10.5 | 10.3 | 2.9 | 8.6 | .0007 |
| Suspected/confirmed systemic infection, % | 4.2 | 5.8 | 8.2 | 5.3 | 3.8 | 1.7 | .239 |
| Female, % | 44.6 | 49.7 | 47.8 | 50.0 | 53.3 | 53.9 | .4835 |
| Race: White, % | 50.3 | 88.3 | 77.4 | 91.0 | 65.4 | 64.1 | <.0001 |
| Race: Black, % | 46.1 | 7.5 | 21.1 | 1.3 | 29.8 | 31.6 |  |
| Ethnicity: Latin-X | 3.9 | 14.8 | 2.3 | 12.8 | 15.4 | 3.4 | <.0001 |
| Medical or social reason for hospitalization | 51.9 | 48.2 | 86.6 | 57.7 | 13.3 | 87.5 | <.0001 |
| Any Cancer | 26.9 | 19.6 | 35.1 | 18.0 | 26.7 | 17.1 | .004 |
| Preceding episode of syncope | 12.2 | 4.2 | 8.9 | 15.4 | 8.6 | 11.1 | .0369 |
| CT RV:LV ratio elevated | 44.8 | 12.9 | 18.7 | 29.5 | 31.4 | 61.5 | <.0001 |
| Echocardiography: RV abnormality | 34.6 | 22.7 | 24.6 | 30.3 | 38.5 | 58.1 | <.0001 |
| Troponin elevation, % | 36.1 | 22.5 | 25.4 | 27.3 | 24.8 | 30.4 | .021 |
| Primary outcome positive, % | 16.0 | 8.5 | 25.4 | 43.6 | 28.6 | 41.0 | <.0001 |

**Abbreviations:** CMC = Carolinas Medical Center, Charlotte, North Carolina; SD = San Diego Medical Center, San Diego, California; VUM = Vanderbilt University Medical Center, Nashville, Tennessee; UU = Utah University, Utah; ORM = Orlando Regional Medical Center, Orlando, Florida; CC = Christiana Care, Newark, Delaware; CT = computed tomography; LV = left ventricle; RV = right ventricle
