# Supplementary material for: Development and validation of a prognostic tool: Pulmonary embolism short-term clinical outcomes risk estimation (PE-SCORE)
Source: PLoS One. 2021 Nov 18;16(11):e0260036. doi: 10.1371/journal.pone.0260036 (PMC8601564; doi:10.1371/journal.pone.0260036)
Supplement: S3 Table — (DOCX) [file pone.0260036.s003.docx]

**Supplemental Table 3: Prognostic Performance of sPESI and ESC at low-risk threshold**

| **Low-risk sPESI**  **on development database** | **Primary**  **Outcome +** | **Primary Outcome -** |
| --- | --- | --- |
| No | 178 (85.2%) | 443 (61.0%) |
| Yes  (sPESI = 0 points) | 31 (14.8%) | 283 (39.0%) |
| sensitivity 85.2% (79.6%–89.7%), specificity 39.0% (35.4%–42.6%), PPV 28.7% (27.0%–30.4%), NPV 90.1% (86.7%–92.8%), and accuracy 49.3% (46.1%–52.6%) | | |

| **Low-risk ESC**  **on development database** | **Primary**  **Outcome +** | **Primary**  **outcome -** |
| --- | --- | --- |
| No | 208 (99.5%) | 650 (89.5%) |
| Yes | 1 (0.5%) | 76 (10.5%) |
| sensitivity 99.5% (97.4%–99.9%); specificity 10.5% (8.3%–12.9%); PPV 24.2% (23.8 -24.7%); NPV 98.7% (91.4%–99.8%), and accuracy 30.4% (27.4%–33.4%) | | |

| **Low-risk sPESI**  **on validation database** | **Primary**  **Outcome +** | **Primary Outcome -** |
| --- | --- | --- |
| No | 172 (80.4%) | 332 (56.6%) |
| Yes  (sPESI = 0 points) | 42 (19.6%) | 255 (43.4%) |
| sensitivity 80.4% (74.4%–85.5%); specificity 43.4% (39.4%–47.6%); PPV 34.1%  (32.0%–36.3%); NPV 85.9% (82.0%–89.0%), and accuracy 53.3% (49.8%–56.8%) | | |

| **Low-risk ESC**  **on validation database** | **Primary outcome +** | **Primary**  **outcome -** |
| --- | --- | --- |
| No | 209 (97.7%) | 486 (82.8%) |
| Yes | 5 (2.3%) | 101 (17.2%) |
| sensitivity 97.7% (94.6%–99.2%); specificity 17.2% (14.2%–20.5%); PPV 30.1% (29.2%–31.0%); NPV 95.3% (89.3%–98.0%), and accuracy 38.7% (35.3%–42.2%) | | |
