# Supplementary figures and images for: Development and validation of a prognostic tool: Pulmonary embolism short-term clinical outcomes risk estimation (PE-SCORE)
Source: PLoS One. 2021 Nov 18;16(11):e0260036. doi: 10.1371/journal.pone.0260036 (PMC8601564; doi:10.1371/journal.pone.0260036)

**Supplemental Figure 1:** Assignment of points to each of the nine variables in the PE-SCORE model


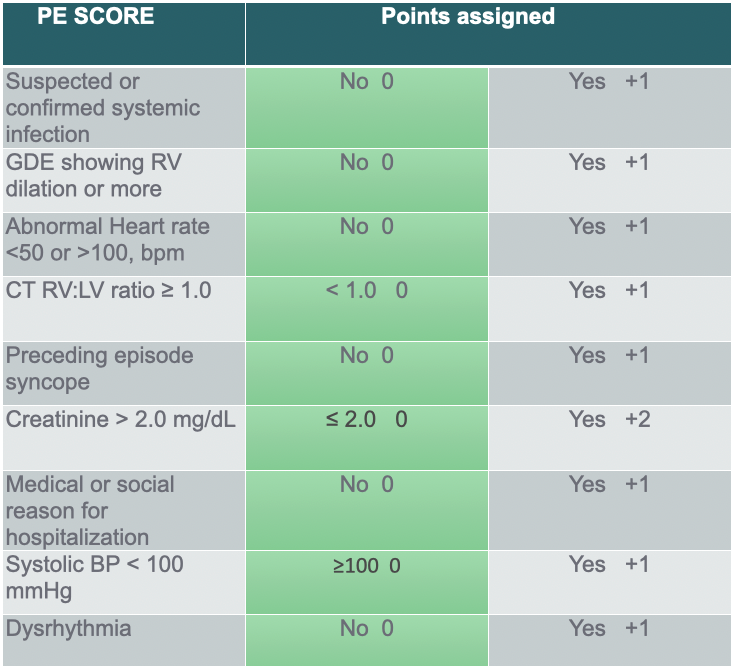

Supplement: S1 Fig — (DOCX) [file pone.0260036.s004.docx]
