# Supplementary material for: Development and validation of a prognostic tool: Pulmonary embolism short-term clinical outcomes risk estimation (PE-SCORE)
Source: PLoS One. 2021 Nov 18;16(11):e0260036. doi: 10.1371/journal.pone.0260036 (PMC8601564; doi:10.1371/journal.pone.0260036)
Supplement: Attachment — Submitted filename: PONE-D-21-19015_revision.pdf [file pone.0260036.s008.pdf]

Revision of the Manuscript:

Development and validation of a prognostic tool: pulmonary embolism short-term clinical outcomes risk estimation (PE-SCORE)

I would like to start by saying that I am not an expert on the pathology in question, but the considerations brought by the authors seem to me to be well argued and with adequate bibliographic references.

The aim of the study is exposed in a very clear way.

I really appreciated the effort to maintain a high degree of rigor in the statistical analysis.

Overall, the study is interesting and well conducted. However, I believe that some methodological aspects can be improved. Below I would like to give some suggestions that I think can further enhance the clarity of the presentation and the strength of the results.

Major comments

A first suggestion is to introduce a header like "Model development" before starting line 226 and to remove the header "Logistic regression", continuing without interruption.

Another suggestion is to move the section "Validation" after the section "Presentation of prediction model:", to keep close the sections related to model development and in turn the sections concerning model evaluation and validation.

A few specific comments:

- line 227: only the parametric t-test is considered. In my esperience, very often biomarkers are affected by even extreme outliers. I suggest to take into consideration the Mann-Whitney and the Wilcoxon tests for variables heavily affected by outliers and more in general for non normal variables
- line 229: the significance level of 5% is very tight for a preliminary analysis. In general, I would suggest to try with a higher threshold, like 10%. Hosmer and Lemeshow[1] suggest the threshold of 25%, for instance, when a univariable logistic regression is considered. In this case, the threshold of 10% would mean, looking at the Supplementary Table 1, the inclusion of NT Pro BNP (p=0.08), while Ethnicity and Recent Trauma would be included by considering 25% as a threshold.
  By the way, in Table 2 I would also indicate the result for binary variables derived from continuous ones (like Age > 80 ys).
- line 229: in Figure 1 it is mentioned that the clinical importance has salso been considered for the preliminary screening of variables. If so, I suggest saying it here.
- line 230: Please, replace "mutivariate" by "multivariable. "Multivariable" is the most commonly used term in the context of regression were a univariate outcome is observed and $p \geq 2$ predictors are considered. Maybe, but I could be wrong, "multivariate" is most used in the context of Machine Learning.
- lines 235-237: Does this mean that the regularizing parameter of the LASSO procedure is chosen by the CV procedure?

[1] Hosmer, D. W., Lemeshow, S. (2004). Applied Logistic Regression. Germany: Wiley.

– line 243: a univariate analysis detecting site differences on key variables is mentioned: more details on this should be provided, considering both applied methods and the type of key variable that was chosed for this analysis. In Supplemental Table 2, the test used for p-value computation should be specified.

However, I found the complex description of the method for model development rather difficult to follow, as the procedure is not entirely standard. Figure 1 was really helpful for me to understand the process, and this leads me to suggest organizing the text from lines 226 to 253 into steps, somewhat mimicking Figure 1. Below I try to propose a reorganization, but obviously the authors should not feel obliged to present it talis et qualis but should only consider it as a suggestion. In my proposal, I'll just consider the text in the manuscript and add further comments *in blue italic* about this text.

Possibile reorganization from line 230

After the preliminary screening:

1. we used a least absolute shrinkage operator (LASSO) logistic regression model for variable selection. *Details about LASSO can be given after the list*
2. We ran standard logistic regression with primary outcome as the response variable on the development dataset and used all variables selected by the LASSO procedure.
3. We excluded predictor variables with $p > 0.05$ then ran the reduced model to create a more parsimonious model. *This is not an efficient way to carry out variable selection in a logistic model. Backward-forward analysis could be considered, for instance, which provides a formal test of the reduced model in comparison with the larger one. It is not unusual that variables with individual p-value >* 0.05 *in the t-test are still retained. For a general discussion, see the previously mentioned book for instance.*
4. To adjust for intra-site clustering of effects, we ran a generalized linear mixed model (GLMM) on the development database with a random intercept term for site. *Lookin at Figure 1 it seems to me that a variable was added to a preliminary GLMM model which was then substituted by another variable. Please, add here a short methodological explanation for this.*
5. *The final model is a logistic model with predictors obtained at the previous step, if I interpret it correctly*

All the comments in between (about LASSO, ROC curves, ecc.) can then be added here, after the list, and even further deepened.

<u>General comments on this five-steps procedure</u>: to the best of my knowledge, the procedure is quite unusual. I would suggest to add a short explanation of why it has been decided to build the model in this way. In particular, it is not clear to me the role of the computation of several ROC curves for intermediate models. It has been commented many times in the literature that this method is not suitable for the selection of variables[2,3]. The ROC curve and related AUC values are very often used to assess the validity of

[2]Hlatky, Mark A., et al. "Criteria for evaluation of novel markers of cardiovascular risk: a scientific statement from the American Heart Association." Circulation 119.17 (2009): 2408-2416.

[3]Pepe MS, Janes H, Longton G, Leisenring W, Newcomb P. Limitations of the odds ratio in gauging the performance of a diagnostic, prognostic, or screening marker. American Journal of Epidemiology 2004; 159(9):882–890

a new biomarker but coupled with an already well assessed model[4]. However, for these purposes as well the likelihood ratio test should be preferred[5,6]. I would therefore like the authors to illustrate their motivations and/or add a few more references.

In my opinion, the session “Validation” should better clarify the two different situations there considered. At the beginning of the session, the use of ROC curves is briefly described with respect to the internal validation of the initially developed model (step 1 in the above step-sequence). In my opinion, this is not relevant for the validation of the final developed model, which is by far of greater interest.

However, no method for external validation is presented. Please, note that lines 263-264 suggest that p-values should be added to Table 1 (the same comment applies to lines 296-297: here the sentence is not supported by tests results). As mentioned before, I would suggest to reorganize the sessions “Validation” and “Model performances” as these two concepts are very related and, in my opinion, slightly confused in the current presentation. For instance, “calibration” is discussed in the session “model performance”, however this issue is mainly related to the validation of a model on a different population.

A few specific comments:

- lines 280-281: Please, add some reference about the area under precision recall curve and the index F1: these terms are most common in Machine Learning than in the traditional, regression-based framework. Moreover, add a few words of explanation about the difference among AUC and AUCpr.
- line 283: please, add a reference for the Spiegelhalter z-test.

The section “Model development” (line 320) discusses the topic in sligthly less detail than Figure 1 shows, in my opinion. For instance, in the text the part related to “other reasons of hospitalization” and “clotting disorder” is not discussed. Please, check the completeness of this section and add a few details about variables selection.

Other specific comments:

- lines 351-352 and Table 3: in the text it is stated that odds ratios of the final nine variables were similar in development and validation cohorts as shown in Table 3. However, Table 3 shows odds ratios for the development cohort and relative risks for the two cohorts. Please, clarify this point.
- line 352: please, clarify why 2 points are assigned to Severe Renal Impairment and 1 point is assigned to any other variable. More specifically, as at line 265 the authors stated “Points assigned were proportional to the odds ratio” and later on (lines 269-271) “we assigned whole points and weights to the final variables of the nine-point tool, which were proportional to each variable’s odds ratio for the primary outcome.” Try also to explain how points have been assigned according to that statements. Finally, where are the weights mentioned and how are they used?
- lines 351-352: again, did you mean that the 9-variables model had also been estimated on the validation cohort? That is, the variables have been kept fixed and regression coeffiecients re-

[4] Xanthakis, Vanessa, et al. "Assessing the incremental predictive performance of novel biomarkers over standard predictors." Statistics in medicine 33.15 (2014): 2577-2584

[5] Vickers AJ, Cronin AM, Begg CB. One statistical test is sufficient for assessing new predictive markers. BMC Med Res Methodol. (2011) 11:13. doi: 10.1186/1471-2288-11-13

[6] Pepe MS, Kerr KF, Longton G, Wang Z. Testing for improvement in prediction model performance. Stat Med. (2013) 32:1467–82. doi: 10.1002/sim.5727

estimated? If I understand correctly, can you confirm that this was done only as a support in deciding the weights of the point model? In this case, however, it seems to me that we are somehow evaluating how the model works in the validation cohort. In other words, it seems a way to also tune the points model on the validation cohort. I'm not sure I understand correctly.

- lines 358-359: why were 95% of the development cohort and 92% of the validation cohort considered? Were these subgroups chosen at random? Lines 402-404 clarify that these are the percentages of subjects with complete records on the variable of interest: I suggest to anticipate here this information.
- line 361 and related Table 4: maybe, an instance of how to compute these values can be provided, for the sake of reproducibility. Either the methods section or the results section should give readers enough information so that they can understand and repeat these analyses. *Please, always consider this rule both when reviewing the methods section and the results section.*
- lines 363-367: this kind of results could be better presented by a barchart like the following

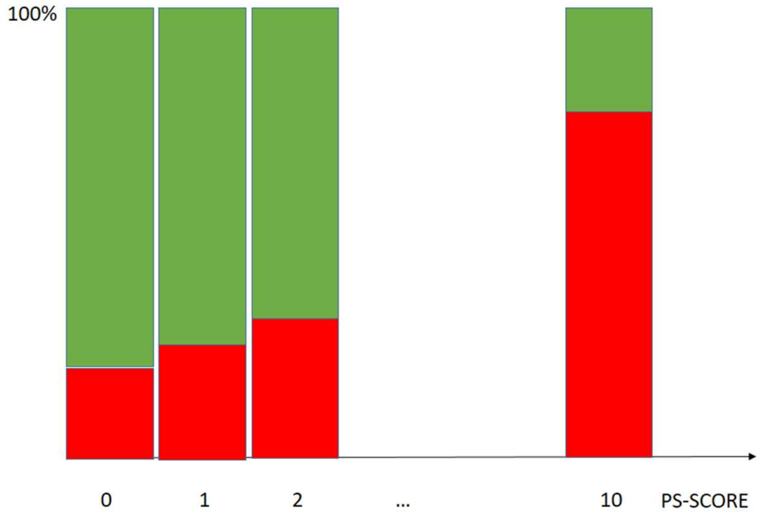

where the red part represents the percentage of primary events and the green part its complement. This is only a suggestion, please feel free to ignore/modify the idea. However, this representation (or any similar one you prefer) has the advantage of immediately shows the relationship between the score and the observed primary events.

- lines 377-378: as in Table 5 several discrimination and calibration metrics are presented, the results for each should be discussed in the text. I also noted that AUC values are compared on a descriptive basis: I suggest to add the p-value of a formal test to increase the strength of the results[7].
- Table 5: in the column of the Spiegelhalter z-test I suggest to write "Spiegelhalter z test *and* p-value": to clarify the meaning for the two values. Alternatively, only the p-value can be indicated as in the case of the Hosmer-Lemeshow test (last column), without changing the text.
- lines 396 and following: here the discussion on the risk thresholds is started, but in lines 406-407 and following the discussion of the performance of the logistic model and of the PE-SCORE is continued. I suggest clearly separating the discussion on the logistics / points model from the

[7] By using http://support.sas.com/kb/45/339.html and http://support.sas.com/kb/25/017.html for instance, to test AUC values in either independent or correlated sample, for instance. Maybe, some test is also available to assess AUCpr values and compare AUCpr values on two (in)dependent samples.

discussion on risk thresholds. Maybe, the discussion of the results presented in Table 5 and Figures 3-4 can be reorganized in an more homogeneous manner.

- lines 409-411: can the p-value of some test be added to these qualitative evaluations? That is, can an evaluation of the statistical significance of a slope > 1 be added? In the logistic regression framework, this can be easily done[8,9]. In the absence of this assessment, it is difficult to establish whether there is a real effect or is it just a sampling issue.
- Table 6: please add the meaning to each letter A, B, C, D.
- lines 429-433: here the results of the Supplementary Table 3 are presented but not discussed. Please, either add a few comments or remove this part.

Minor comments

- line 27: Add the acronym "(RV)" here instead of line 29.
- line 32: Please, substitute here *predictive* with *prognostic*, to make immediately clear what are you interested in predicting.
- line 97: here 107 "data elements" are mentioned while at line 138, 138 variables are mentioned: could you better clarify this point, please?
- line 148-149: It is not clear to me what is meant by the sentence about sensitivity analysis.
- line 192: I have really appreciated this subsection! I have very rarely found sample size estimates in studies like this one.
- line 251: as the analysis based on the random forest approach is not presented in the section Results, but only briefly discussed in the Discussion section, I suggest to avoid any mention here.
- line 276 and in the following: maybe "PE-SCORE" can be simply used without to continuously mention "points model". Il seems to me that the good acronym "SCORE" is well self-explaining.
- line 302: Once again, it is not clear to me what is meant by the sentence about sensitivity analysis. According to what I mean by the term "Sensitivity analysis", the comparison of frequencies in different groups is not sensitivity analysis.
- line 323: please add "($p > 0.25$)" at the end of the comment about cancer and heart failure. In general, add the corresponding p-values or a meaningful summary of them in the text, to facilitate the reader and directly support the statements.
- line 345: Section "Model specification" can be removed, in my opinion, without loss of relevant information.
- Table 6: Recall=A/(A+B)
- lines 478-79: please, add the references of the 5 studies mentioned here; add also the references of the few studies including images as candidate variables.

---

[8] Genders TS, Steyerberg EW, Alkadhi H, Leschka S, Desbiolles L, Nieman K, et al. A clinical prediction rule for the diagnosis of coronary artery disease: validation, updating, and extension. Eur Heart J. (2011) 32:1316–30. doi: 10.1093/eurheartj/ehr014

[9] Moons, Karel GM, et al. "Risk prediction models: II. External validation, model updating, and impact assessment." Heart 98.9 (2012): 691-698.
