# Supplementary material for: Development and validation of a prognostic tool: Pulmonary embolism short-term clinical outcomes risk estimation (PE-SCORE)
Source: PLoS One. 2021 Nov 18;16(11):e0260036. doi: 10.1371/journal.pone.0260036 (PMC8601564; doi:10.1371/journal.pone.0260036)
Supplement: Attachment — Submitted filename: Response to Reviewers.docx [file pone.0260036.s009.docx]

August 25, 2021

Robert Ehrman, MD, MS

Academic Editor, PLOS One

RE: PONE-D-21-19015

Development and validation of a prognostic tool: pulmonary embolism short-term clinical outcomes risk estimation (PE-SCORE)

Thank you and the reviewers for the very helpful comments and suggestions. We have responded in detail using red font to each observation or suggestion of the reviewers. We believe the revisions have made the report clearer and better for readers. We are pleased to submit our revised manuscript, which now includes new text, data, and figures. In the “track changes” version of our enclosed manuscript, red font denotes additions to the original submission, while strikethrough text denotes deletions to the original. We hope our resubmission meets with full acceptance.

Thank you for considering our work.

Best regards,

Anthony J. Weekes, MD, MSc

On behalf of the PESCOR Steering Committee

PONE-D-21-19015

Development and validation of a prognostic tool: pulmonary embolism short-term clinical outcomes risk estimation (PE-SCORE)

PLOS ONE

**ACADEMIC EDITOR COMMENTS:**

I applaud the authors for undertaking this large and important task--the need for a tool to help stratify patients for short-term adverse outcomes in PE is sorely needed, as is incorporation of an acute measure of RV function on echocardiography.

In addition to the comments by the reviewers, please consider the following when preparing a revision.

-It appears that acute and chronic RV dysfunction were treated equally, but i am not certain this is appropriate, since the goal of the paper is to include PE-related RV dysfunction (RVD). For example, patients with RVD from pre-existing post-capillary pHTN may have outcomes different from those with pre-existing RVD from pre-capillary pHTN which may also be different from those with acute onset pHTN from a PE--is it the RVD that leads to the bad outcome or is it the underlying lung or heart disease? Compounding this is the fact that size and location of PE are not reported, which potentially further distorts the relationship between the PE, RV function, and outcomes (particularly since PE severity is emphasized in the discussion). i would be interested in seeing an analysis of only those with acute RVD, as i suspect that patients with chronic RVD have a greater likelihood of poor outcomes at regardless of PE size.

We appreciate both concerns and therefore include the following explanations and additional data.

The goal was to detect the presence or absence of specific RV abnormalities. We had guidelines to suggest acute versus long standing RV abnormalities. Clinical context, RV wall thickness and co-existing or preexisting echocardiography findings if available would help distinguish if RV abnormalities were acute, chronic, or indeterminate.

We now include reports for systolic blood pressure < 100 mmHg, age < 80 years, abnormal heart rate (< 50 or > 100 beats/min), when GDE is considered abnormal, is the RV abnormality acute, chronic or indeterminate, and most proximal location of clot on computed tomography pulmonary angiography (saddle proximal portion of pulmonary arteries, lobar, segmental, or subsegmental). These above-mentioned variables are now included with descriptive and univariate statistics of Table 1, Table 2, and Supplemental Table 1.

-I wonder about the extent to which the presence of sepsis accounts for some of the adverse outcomes as opposed to the PE? PE is often accompanied by co existing medical condition.

Our variable is suspected or confirmed sepsis/co-existing infection. Conditions associated with suspected or proven infection may lead to immobility and development of PE or susceptibility to added insults from PE. However, the PE itself may be relatively small. An affirmative response for this variable in our database was uncommon. Interactions between clinical condition variables are inevitable. For example, cancer can provoke PE and may or may not be associated with some of the adverse outcomes. This is a statistical inference report and not a report of causal relationships. It is plausible that co-existing left ventricular dysfunction may be associated with some of the adverse outcomes in PE or hamper a compensatory response.

For validated PE prognostic models like PESI, SPESI and Hestia, it is plausible that predictors in the model (e.g., cancer and cardiopulmonary disease) may be associated with the outcomes reported.

-line 467-469: this does not really make sense to me as the putative pathway for right heart dysfunction leading to adverse outcomes is via reduced left heart function from reduced right-sided output. in addition, i think this statement needs a reference. also, can you include the number of patients that had troponin and BNP levels drawn?

BNP reflects on myocardial stretch and is used in congestive heart failure, acute coronary syndrome, myocardial infarction, and PE. Troponin reports on myocardial injury. Neither of these biomarkers identify which of the cardiac chambers is stretched or injured as well as imaging. The specificity of BNP and troponin are poor compared to RV enlargement or RV systolic dysfunction as determined by comprehensive echocardiography. Poor LV filling and poor LV output are provoked by compromised and delayed RV emptying, decreased RV systolic function, leftward septal shift, and reduced LV cardiac output due to underfilling.

We now have reports of the number of patients with laboratory measurements (coded both as continuous and categorical variables), including missingness, in Table 1, Table 2, and Supplemental Table 1.

-while the NPV point estimate is quite high, the lower end of the CI may be too low for some providers to feel safe discharging these patients--can you discuss this?

This study addresses hesitancy of point-of-care providers even when using validated prognostic models like PESI and sPESI. We do so by directly addressing concerns of missing underlying RV abnormalities and focusing on other outcomes that matter to providers but are not addressed by those models. Recent prognostic model and practice pathways have added more assessments beyond those used in validated clinical derived prognostic models.^1-2^

If this prognostic model is validated or updated, we believe the next steps will impact studies to evaluate its influence on clinical decision making as alluded to.

Active comorbid conditions may adversely affect the outcomes of PE patients. In this study, the patient with PE-SCORE of zero had segmental PE [no RV abnormality on imaging (CT or GDE)], did not receive anticoagulation, and had immediate complications of perforated gastrointestinal ulcer. We did not distinguish PE-related death from death within 5 days.

^1^ [Singer AJ, Thode HC Jr, Peacock WF th. Admission rates for emergency department patients with venous thromboembolism and estimation of the proportion of low risk pulmonary embolism patients: a US perspective. *Clin Exp Emerg Med* 2016;**3**:126–131.](http://paperpile.com/b/P4FRKo/8iye)

^2^ [Vinson DR, Ballard DW, Huang J, Reed ME, Lin JS, Kene MV, Sax DR, Rauchwerger AS, Wang DH, McLachlan DI, Pleshakov TS, Silver MA, Clague VA, Klonecke AS, Mark DG, Maple Investigators of the KP CREST Network. Outpatient Management of Emergency Department Patients With Acute Pulmonary Embolism: Variation, Patient Characteristics, and Outcomes. *Ann Emerg Med* 2018;**72**:62–72 e3.](http://paperpile.com/b/P4FRKo/oBK9)

-was there any measurement of inter-rater reliability or central adjudication of RV findings on echo?

We used a criteria grading system with high inter-rater reliability (kappa = 0.84) for severe RV dilatation between emergency physicians and cardiologists.^1^ Quality assurance reviews were performed by experienced clinical ultrasound leaders. One challenge with comparing comprehensive echocardiography studies is that descriptors of severe to moderate RV dilatation or systolic function were not standardized by the American Society of Echocardiography.

^1^ Weekes AJ, Oh L, Thacker G, Johnson AK, Runyon M, Rose G, Johnson T, Templin M, Norton HJ. Interobserver and intraobserver agreement on qualitative assessments of right ventricular dysfunction with echocardiography in patients with pulmonary embolism. *Journal of Ultrasound in Medicine: Official Journal of the American Institute of Ultrasound in Medicine* 2016; 35(10): 2113–20.

-feasibility of GDE is really outside the scope of this paper and i would suggest removing this section from the discussion.

We appreciate this view. We also note another reviewer's comment expressing concern about the feasibility of goal directed echocardiography. We think a de facto disclosure of one of the variables in the study, which identified the level of clinical experience of providers performing goal directed echocardiography, in the results section is pertinent to this report. We will remove further discussion about the feasibility of GDE in the discussion.

-overall, the statistical methods are quite robust. i would consider, however, moving some of the technical details to a supplement as i suspect that many readers who will find this paper most interesting will be clinicians for whom the statistical details might be overwhelming/distracting.

We will do as you suggest. One reviewer offered a great suggestion for an outline of key steps before providing details that we think would help.

**REVIEWER COMMENTS TO THE AUTHOR:**

**Reviewer #1:** Interesting paper.Some issues, although methodological aspects are very strong and accurate.

1) Deterioration should be better defined in abstract and in paper.

Yes, you are correct. ‘Deterioration’ and it is frequently used without a clear definition. This was a huge oversight.

In response, we concisely explain ‘deterioration’ in the abstract, introduction, and in the outcomes section as follows. ”The primary composite outcome was death and early deterioration (respiratory failure, cardiac arrest, new dysrhythmia, sustained hypotension, and rescue reperfusion intervention) within 5 days.”

2) Abstract: CI for AUC should be added

We added AUC logistic regression development 0.83 (0.80, 0.86)

AUC PE-SCORE development 0.78 (0.75, 0.82); validation 0.77 (0.73, 0.81)

AUC pr development 0.61 (0.57, 0.64)

AUC pr PE-SCORE development 0.50 (0.39, 0.60); validation 0.63 (0.43, 0.81)

3) methods: Authors stated that they performed sensitivity analysis for each candidate. This should be better specified

We will correct this statement to indicate we performed a sensitivity analysis for the final model predictors with the most missingness.

In the Results section, we now include the following statements: Twenty-one (2.2%) of the patients in the developmental database were missing GDE. Most of these missing GDE scores were marked as inadequate for interpretation. Six of the 21 had a positive primary outcome. The percentages of patients experiencing the primary outcome among those with GDE showing RVD negative, positive, and missing were 14.4%, 38.4%, and 28.6%, respectively.

4) Data cleaning is not clear

Here is the revised data cleaning section:

During the enrollment phase, important variables were assessed for missingness and discrepancies during data cleaning. For example, we reported outliers in vital signs or laboratory measurement values to the site investigators. At the close of enrollments, descriptive statistics were used to examine predictor and outcome variables for sensibility and missiness. Instructions for corrective actions were assigned to the site investigator and clinical research team by referring to source documents within the electronic health records. After missingness was mitigated and sensibility of data optimized, the database was used for analysis.

**Reviewer #2: Overall**

This paper represents a valiant effort to create a new discriminating decision-making tool for patients with pulmonary embolism including RV echo findings as a variable. There is a need for PE management decision-making tools that consider this variable. Overall though, this tool does not appear to reliably perform at the high level of some of the existing decision-making tools and therefore its utility in practice is at this time limited. Perhaps if validated in a larger cohort of patients outside of one of the original participating institutions it might strengthen the argument for the utility of this decision-making tool but as it stands this tool’s performance appears mediocre. The concept of a risk stratifying PE tool is not novel as there are several other well validated decision-making tools in use for determining low vs high risk PE. PESI, HESTIA, ESC and sPESI consistently have very good, reliably reproducible negative predictive value in multiple validation cohorts and do not require users to have any ultrasound training.

The report is focused on the information provided by early assessments of cardiac biomarkers and readily available imaging by echocardiography and CT. Outcomes of death are becoming less common. Bleeding and recurrent VTE are less common with proper screening of bleeding risks, and with the current safety and efficacy profiles of novel anticoagulation agents. A related but separate discussion is the skill set of echocardiography, which is an expected procedural skill with milestones at accredited emergency medicine residency training programs. The prognostic model we report on addresses a composite of clinical outcomes that are not addressed by the negative predictive values for the above mentioned PESI, Hestia, ESC, and sPESI. The meta analysis by Barco et al. demonstrated the added prognostic value of RV assessments by biomarkers or imaging over PESI/sPESI alone for the outcome of death and this was corroborated by another meta-analysis in 2021 (Beccatini et al.).

While it is unique (and likely important) to include POCUS RV abnormalities as a variable in the tool and there is some literature that supports including this variable in the determination of low vs high risk PE ([https://academic.oup.com/eurheartj/article/40/11/902/5263773](https://urldefense.com/v3/__https:/academic.oup.com/eurheartj/article/40/11/902/5263773__;!!Kv7QgGdTlhIaqSqT!dbOz7_zlC6ES5FPkOY4euzcNRjKrK7gVVPI8rh6fp_gPS097Nc5c0Ajzu-CUyFMvui6eBA$)), inclusion of this variable further limits the generalizability of this tool as there are still a great number of EM trained physicians and midlevel providers who are not POCUS trained or are only minimally pocus trained. Were this tool to demonstrate exceptional discrimination with the addition of the RV echo findings variable there would be an argument for using it however, it underperforms and is more complex to use as compared to existing tools.

This prognostic model is not complex. It uses readily available information at the point of care.

Fifteen years ago, the stated premise of seeking surrogate markers of adverse events in normotensive PE was the unavailability of echocardiography for risk stratification.”Although echocardiography has proven utility in risk stratifying normotensive patients with pulmonary embolism, echocardiography is not always available. OBJECTIVE: Test if a novel panel consisting of pulse oximetry, 12-lead electrocardiography, and serum troponin T would have prognostic equivalence to echocardiography.”^1^ Although *comprehensive* echocardiography may still not be available at all times, the past decade has seen deliberate incorporation of goal directed echocardiography into the education and practice of acute care providers in emergency medicine and critical care.

^1^ Kline JA, Hernandez-Nino J, Rose GA, Norton HJ, Camargo CA Jr. Surrogate markers for adverse outcomes in normotensive patients with pulmonary embolism. *Critical Care Medicine* 2006; 34(11): 2773–80.

As it stands, this paper represents a good idea that needs further reliably reproducible results (particularly in terms of its negative predictive value for low risk PE) before it would be a novel and worthwhile contribution to the current body of evidence of risk stratifying patients with PE. The result differences between the database and validation groups in some key areas (differences in false negatives between the database and validation group and the underpredicting of the tool for the outcome of interest at all scores 0-9) make this tool at face value seem to perform unreliably. Where it may be somewhat novel (but still requires further validation) is as a risk stratification tool for short term outcomes specifically for patients with ESRD and a PE.

We want to clarify the prognostic model is meant for the undifferentiated PE patient (“all comers'') and not created for moderate to renal disease (defined as creatinine > 2.0, not as ESRD) or co-existing features of systemic infectious illness, suspected/confirmed systemic infection, or cancer. By statistical inference, the model development identified renal disease, concerns for co-existing infection, vital sign abnormalities, and imaging features of RV abnormality amongst 9 variables with higher odds ratio for the outcome of interest.

Because we were not clear enough, we have replaced “renal impairment” with “creatinine greater than 2.0 mg/dL” in tables and main manuscript text.

Abstract

Line 17: This statement is particularly misleading since the validation group had an 8% proportion of the primary outcome in the zero score group.

Thank you for pointing this out. We changed the statement as follows: “As points increased, outcome proportions increased: a score of zero had 2% outcome, whereas scores of six and above had ≥ 69.6% outcomes. In the validation dataset, PE-SCORE zero had 8% outcome [no deaths], whereas all patients with PE-SCORE ≥ 6 had the primary outcome.”

Intro

Well written. Identifies the need for a decision tool that considers RV echo findings. Supports the need for a decision tool that can assess risk of short term deterioration and not risk of 30 day deterioration.

Methods

Enrollment criteria appropriate.

This study is aided by the diversity of its multiple institutions.

Line 110-112, 121-124: Using the need for volume expansion or pressors for documented hypotension as a marker of deterioration from PE may be confounded by the fact that your score includes septic patients. Worsening sepsis may cause the same deterioration which in the model of this study may be incorrectly attributed to the PE when really the hypotension is from the infection.

We incorrectly summarized the variable as ‘sepsis’ in the Tables. The actual variable definition used throughout the study enrollment and data extraction was ‘suspected or confirmed systemic infection’.  Few patients had ‘suspected or confirmed systemic infection.’ This was the meaning of the variable explained during investigator/abstractor teaching and REDCap training sessions.

We have a large sample that represents real world conditions. PE patients have co-existing conditions, such as cardiopulmonary disease and cancer, that may influence vital signs, susceptibility to PE, or response to treatment. We want to clarify that the prognostic model is meant for the undifferentiated PE patient and not created for moderate to severe renal disease or co-existing features of systemic infectious illness or cancer. By statistical inference, the model development identified creatinine 2.0 mg/dL, concerns for co-existing infection, vital sign abnormalities, and imaging features of RV abnormality amongst 9 variables as having higher odds ratio for the outcome of interest.

Methods for selection of variables seems appropriate.

Line 169-177: This change is unfortunate and as you cover in your discussion, may be a confounder.

Yes, it caused mild chagrin.

Sample size calculations are appropriate. Thank you.

Blinding to the development of different databases is a strength. Thank you.

Line 232: Explanation of LASSO is appreciated and helpful to the reader.

Overall, this section is too long and in places too complex. As written, some of it is difficult to understand due to the use of some statistics that are not frequently encountered (ex Line 283).

May benefit from a simpler explanation of why these tests were utilized. This comes up again in the data section with the use of F1 and Hosmer-Lemshow. Statistics chosen do seem appropriate for the data set and aim of the study.

We provide a comprehensive report of recommended measurements of calibration. We include support for other assessment of calibration, including one that discounts the reliance on Hosmer Lemeshow to assess and prove calibration.^1-2^ Calibration slope and intercept can have confidence intervals (see Table 4 in the article referenced).^2^ When slope (spread) and intercept are reported together, a slope close to 1 and intercept close to 0 provide good commentary on calibration.

^1^ Van Calster B, McLernon DJ, van Smeden M, Wynants L, Steyerberg EW. Calibration: the Achilles heel of predictive analytics. *BMC Medicine 2019;* 17(1): 230. https://doi.org/10.1186/s12916-019-1466-7

^2^ Walsh, Colin G., Kavya Sharman, and George Hripcsak. 2017. “Beyond Discrimination: A Comparison of Calibration Methods and Clinical Usefulness of Predictive Models of Readmission Risk.” *Journal of Biomedical Informatics* 76 (December): 9–18.

Results

Hosmer-Lemeshow for the regression model development database and particularly the validation database indicates poor fit.

We provide 3 other recommended calibration metrics and include Hosmer Lemeshow, with references supporting use of other calibration metrics over the Hosmer Lemeshow given its limitations.

F1 score for the high-risk pool seems poor given that this group should be skewed to see more subjects with the primary outcome (I’d assume individuals with a high risk score should experience an increased number of poor outcomes).

Confidence interval for the PPV of the high-risk group is quite wide so while the reported PPV looks good the certainty that it is actually close to 90% is not great (according to that confidence interval it could be less than 80% which at that point, how much utility does this test have?).

It appears the tool significantly underpredicted the primary outcome at every score level in the validation group, particularly in the categorized low risk groups (0-4), which is concerning if this tool is being advertised to discriminate between patients that have a high likelihood of deterioration and require admission vs low likelihood of deterioration and suitability for outpatient management (unless you plan on advertising this tool as dichotomous (less than or equal to zero is low risk, anything else isn’t)).

We report on the model’s performance in low versus high risk stratification because the decisions made are quite different. Low risk stratification increases consideration for immediate outpatient clinical management, whereas high risk stratification increases the intensity of monitoring.

While the NPV in the low-risk database group with the cut-off set to “0” looks great (NPV 97.9% (93.6 -99.5%)) it underperforms in the validation group (91.9%). Some of the existing PE tools consistently perform at a NPV >97% over multiple validation studies. The PPV of the high-risk PE threshold group appears to suffer similarly where it performs well in the validation group but suffers in the database group. plos

That is the point of the proposal. WIth important outcomes in mind besides death, what are the predictors that providers at the point of care should consider to optimize risk stratification both at the low end and the higher threshold? sPESI/PESI, Hestia, and ESC have different components, different outcomes, different performances, and are perhaps not interchangeable when applied to undifferentiated PE patients.

PE-SCORE was created for the described primary outcome and considered a large number of candidate variables that are readily available at the point of decision making.

Our process for developing a prognostic model selected renal disease (creatinine > 2.0 mg/dL ) as one of the final components by statistical inference. PE-SCORE was not developed to target patients with cardiac disease or renal disease, just as sPESI and Hestia were not developed for patients with cancer, cardiopulmonary disease, low blood pressure, or fast heart rates. It identified the presence or absence of creatinine > 2.0 mg/dL, vital sign abnormalities, and RV abnormalities as important criteria in estimating graded probability of developing the defined outcome.

408-414: Though several tests indicate good calibration, your data shows this tool underpredicted the primary outcome at every score level.

Although prognostic models are often wrong, some are useful. Our primary composite outcome is not simple and some may even consider it stringent. We did not look for a single important yet uncommon primary outcome like death. We had very stringent criteria with a primary composite outcome (death, hypotension involving pressors, respiratory insufficiency, etc.). We believe external validation would provide an opportunity to improve the model.

Discussion

While the AUC values for this tool are fair to good, the AUC precision recall values are not which is reflected in the less than robust PPV values for this score at the high threshold.

Lines 462-470: This is definitely an interesting thought.

Line 483-485: Sums up why this score (if proven to be more reliable in the future) would be a clinical difference maker. Thank you. We think this is useful.

Line 492-494: This is exactly what I believe needs to happen to make this more publishable.

We developed a prognostic model and performed validation. The opportunity for others to perform external validation, update the model, or conduct an impact study on prognosis or clinical decision making of the model would be beneficial.

Line 505-506: Agree.

Line 548-550: Agree and this should be a separate publication from this dataset.

Conclusion

Line 577-579: Depends on what you are considering a cutoff for low or high probability. Having clinical deterioration occur within 5 days in 8% of the zero score individuals of the validation group seems uncomfortably high when many of our other decision tools set the threshold at 2% (which was seen in the database group).

The primary composite outcome is defined as one or more of specific predefined clinical deterioration events described in the ‘Outcome measures’ subsection within Materials and Methods section. Had we used mortality as the primary outcome, the proportion with the primary outcome would be very low, but we would have excluded *outcomes that matter to those making decisions at the point of care.* We want users to recognize the outcomes that are important.

We took on the risks of having stringent primary composite outcomes and the results may be viewed within that context.

**Reviewer #3:**

Revision of the Manuscript:

Development and validation of a prognostic tool: pulmonary embolism short-term clinical outcomes risk estimation (PE-SCORE)

I would like to start by saying that I am not an expert on the pathology in question, but the considerations brought by the authors seem to me to be well argued and with adequate bibliographic references.

The aim of the study is exposed in a very clear way.

Thank you.

I really appreciated the effort to maintain a high degree of rigor in the statistical analysis.

Thank you.

Overall, the study is interesting and well conducted. However, I believe that some methodological aspects can be improved. Below I would like to give some suggestions that I think can further enhance the clarity of the presentation and the strength of the results.

Thank you. We appreciate any opportunity to make the report better and more clear for readers.

Major comments

A first suggestion is to introduce a header like “Model development” before starting line 226 and to remove the header “Logistic regression”, continuing without interruption.

We appreciate and agree with this suggestion, We made the adjustment.

Another suggestion is to move the section “Validation” after the section “Presentation of prediction model:”, to keep close the sections related to model development and in turn the sections concerning model evaluation and validation.

Correct again. We made the suggested adjustment.

A few specific comments:

− line 227: only the parametric t-test is considered. In my experience, very often biomarkers are affected by even extreme outliers. I suggest to take into consideration the Mann-Whitney and the Wilcoxon tests for variables heavily affected by outliers and more in general for non normal variables

We used Student’s t-test for univariate comparisons of all continuous variables. The t-test does not require an assumption of normally distributed data with sufficiently large samples due to the central limit theorem. The smallest outcome group sample size in our study exceeds 200, which is considered “sufficiently large” for this purpose (Ref: Lumley T, Diehr P, Emerson S, Chen L. The importance of the normality assumption in large public health data sets. Annu. Rev. Public Health 2002; 23:151-169).

− line 229: the significance level of 5% is very tight for a preliminary analysis. In general, I would suggest to try with a higher threshold, like 10%. Hosmer and Lemeshow^1^ suggest the threshold of 25%, for instance, when a univariable logistic regression is considered. In this case, the threshold of 10% would mean, looking at the Supplementary Table 1, the inclusion of NT Pro BNP (p=0.08), while Ethnicity and Recent Trauma would be included by considering 25% as a threshold. By the way, in Table 2 I would also indicate the result for binary variables derived from continuous ones (like Age > 80 ys).

^1^ Hosmer, D. W., Lemeshow, S. (2004). Applied Logistic Regression. Germany: Wiley.

Thank you for this observation. When we used the cut-off of p < 0.05, many continuous and categorical variables had significant differences. This is likely due to the large sample size. The rule for retaining variables was not simply p < 0.05. Rather, the decision whether to retain a variable was based on a combination of factors, including strength of association, prior research findings, and clinical importance.

In addition, although biomarkers (when expressed as continuous variables) may have had p values above the 0.05 cutoff, the p values of the biomarkers coded as categorical variables did have p values below 0.05 as shown in Table 2. The categorical coded variables for troponin and natriuretic peptide elevation were included in the LASSO regression but were not statistically significant by this regression test.

We have now included binary variables derived from continuous variables in Table 2 and Supplementary Table 1.

We now include reports for:

1) Systolic blood pressure (sbp) < 100 mmHg,
2) Age < 80 years,
3) Abnormal heart rate (< 50 or > 100 beats/min),
4) GDE considered abnormal, is RV dilatation acute, chronic, or indeterminate?
5) Most proximal location of clot on CTPA (saddle proximal portion of pulmonary arteries, lober, segmental, or subsegmental).

These categorical variables are now reported with descriptive and univariate statistics in Table 1, Table 2, and Supplemental Table 1.

− line 229: in Figure 1 it is mentioned that the clinical importance has also been considered for the preliminary screening of variables. If so, I suggest saying it here.

We will include the following statement: “Investigators also factored in the clinical importance of statistically significant candidate variables.”

− line 230: Please, replace “multivariate” by “multivariable. "Multivariable" is the most commonly used term in the context of regression were a univariate outcome is observed and *p* ≥ 2 predictors are considered. Maybe, but I could be wrong, "multivariate" is most used in the context of Machine Learning.

We replaced ‘univariate’ with ‘univariable’ and replaced ‘multivariate’ with ‘multivariable’ throughout the manuscript.

− lines 235-237: Does this mean that the regularizing parameter of the LASSO procedure is chosen by the CV procedure?

Yes, that is correct. The regularization parameter for the LASSO procedure was chosen using cross validation. The optimal value is the one that minimizes the cross-validated average squared error.

− line 243: a univariate analysis detecting site differences on key variables is mentioned: more details on this should be provided, considering both applied methods and the type of key variable that was chosen for this analysis. In Supplemental Table 2, the test used for p-value computation should be specified.

To identify candidate variables with plausible correlations with clinical sites for prognostic importance, we performed comparisons between clinical research sites using one-way ANOVA for continuous variables, the Kruskal-Wallis test for ordinal variables, and the chi-square test for categorical variables. Supplemental Table 2 shows there were statistically significant differences between sites for the occurrence of primary composite outcome, race, age, ethnicity, abnormal heart rate, renal impairment, and abnormal RV by imaging. We also compared sites by Charlson score, LV dysfunction, and medical or social need for hospitalization variables. We have added these details to the revised manuscript (text and Supplemental Table 2 footnote).

However, I found the complex description of the method for model development rather difficult to follow, as the procedure is not entirely standard. Figure 1 was really helpful for me to understand the process, and this leads me to suggest organizing the text from lines 226 to 253 into steps, somewhat mimicking Figure 1. Below I try to propose a reorganization, but obviously the authors should not feel obliged to present it talis et qualis but should only consider it as a suggestion. In my proposal, I’ll just consider the text in the manuscript and add further comments in blue italic about this text.

Possibile reorganization from line 230

We like this suggestion and we used this approach. Thank you.

After the preliminary screening:

1. we used a least absolute shrinkage operator (LASSO) logistic regression model for variable selection. Details about LASSO can be given after the list

2. We ran standard logistic regression with primary outcome as the response variable on the development dataset and used all variables selected by the LASSO procedure.

3. We excluded predictor variables with p > 0.05 then ran the reduced model to create a more parsimonious model. This is not an efficient way to carry out variable selection in a logistic model. Backward-forward analysis could be considered, for instance, which provides a formal test of the reduced model in comparison with the larger one. It is not unusual that variables with individual p value > 0.05 in the t-test are still retained. For a general discussion, see the previously mentioned book for instance.

We reviewed your recommendation. We chose to use statistical significance and clinical importance as criteria for further reducing the model to develop a parsimonious model for clinical utility. The LASSO procedure yielded 13 variables, which we ran in a subsequent logistic regression to further assess these variables. Variables with p > 0.10 were removed from further analysis. Based on this logistic, we excluded 4 additional variables.

We did not want to do a stepwise procedure in addition to the LASSO, as we did not want to perform two formal variable selection procedures, and the LASSO has been shown to be superior. We have a frequently cited reference that shows superior performance of the LASSO and a few other techniques for variable selection to traditional stepwise backward elimination methods: Yuan, Ming, Yi Lin. Model selection and estimation in regression with grouped variables. *Journal of the Royal Statistical Society. Series B, Statistical Methodology* 2006; 68(1): 49–67.

To verify our response above, during the ‘revise and reconsider’ period allowed for remitting itemized responses, we ran a stepwise procedure using the 13 variables from the LASSO. With stringent criteria applied (entry and stay criteria of 0.05 each), the resulting model consisted of 8 variables, all of which were in our reduced model. The only variable from our reduced model missing from the stepwise results was rv_lv_ratio_1_0.

4. To adjust for intra-site clustering of effects, we ran a generalized linear mixed model (GLMM) on the development database with a random intercept term for site. Looking at Figure 1 it seems to me that a variable was added to a preliminary GLMM model which was then substituted by another variable. Please, add here a short methodological explanation for this.

This was not done to further reduce the model. This was done to further identify variables with correlations to the clinical site variable. We looked for reasons for differences between clinical sites and determined there were significant differences between sites in the proportion of those considered to have medical or social reasons for hospitalization.

5. The final model is a logistic model with predictors obtained at the previous step, if I interpret it correctly

The final model is a 9-variable logistic regression (LR) prognostic model. Computation of the probability of the outcome requires the logistic regression equation we provided. To facilitate real-time clinical use by providers, we simplified the final 9-variable LR model to a 9-variable points model called PE-SCORE. PE-SCORE has points assigned to each of the 9 variables based on the adjusted odds ratios of the variables in the final model: 0 or 1 point for 8 of the final variables; 0 or 2 points for the absence or presence of renal impairment. We report on the prognostic performance of both forms of the prognostic model. With all 9 variables present for evaluation, the minimum score for PE-SCORE is 0 and the maximum score is 10.

All the comments in between (about LASSO, ROC curves, ecc.) can then be added here, after the list, and even further deepened.

General comments on this five-steps procedure: to the best of my knowledge, the procedure is quite unusual. I would suggest to add a short explanation of why it has been decided to build the model in this way. In particular, it is not clear to me the role of the computation of several ROC curves for intermediate models. It has been commented many times in the literature that this method is not suitable for the selection of variables ^2,3^. The ROC curve and related AUC values are very often used to assess the validity of a new biomarker but coupled with an already well assessed model^4^. However, for these purposes as well the likelihood ratio test should be preferred 5^,6^. I would therefore like the authors to illustrate their motivations and/or add a few more references.

^2^ Hlatky, Mark A., et al. "Criteria for evaluation of novel markers of cardiovascular risk: a scientific statement from the American Heart Association." Circulation 119.17 (2009): 2408-2416.

^3^ Pepe MS, Janes H, Longton G, Leisenring W, Newcomb P. Limitations of the odds ratio in gauging the performance of a diagnostic, prognostic, or screening marker. American Journal of Epidemiology 2004; 159(9):882–890

^4^ Xanthakis, Vanessa, et al. "Assessing the incremental predictive performance of novel biomarkers over standard predictors."

Statistics in medicine 33.15 (2014): 2577-2584

^5^ Vickers AJ, Cronin AM, Begg CB. One statistical test is sufficient for assessing new predictive markers. BMC Med Res Methodol.

(2011) 11:13. doi: 10.1186/1471-2288-11-13

^6^ Pepe MS, Kerr KF, Longton G, Wang Z. Testing for improvement in prediction model performance. Stat Med. (2013) 32:1467–82.

doi: 10.1002/sim.5727

We will explain it better in the manuscript. We developed the final logistic regression prognostic model. To present the final prognostic model in a more clinician-friendly format to improve usefulness, we converted the logistic regression model to a points model (PE-SCORE). Next, we report on the performances of the logistic regression and PE-SCORE prognostic models on the development database with ROC curves.

These are not intermediate models. Rather, ROC curves were created for 1) the logistic regression model, which includes the final set of predictor variables, in the development database, and 2) the total points model in the development database and the validation database.

In my opinion, the session “Validation” should better clarify the two different situations there considered. At the beginning of the session, the use of ROC curves is briefly described with respect to the internal validation of the initially developed model (step 1 in the above step-sequence). In my opinion, this is not relevant for the validation of the final developed model, which is by far of greater interest.

However, no method for external validation is presented. Please, note that lines 263-264 suggest that p values should be added to Table 1 (the same comment applies to lines 296-297: here the sentence is not supported by test results). As mentioned before, I would suggest reorganizing the sessions “Validation” and “Model performances” as these two concepts are very related and, in my opinion, slightly confused in the current presentation. For instance, “calibration” is discussed in the session “model performance”, however this issue is mainly related to the validation of a model on a different population.

We rearranged the sections to make the steps clear and to distinguish internal validation during the prognostic model development from external validation of PE-SCORE, which was performed on a different cohort of PE patients.

A few specific comments:

− lines 280-281: Please, add some reference about the area under precision recall curve and the index F1: these terms are most common in Machine Learning than in the traditional, regression based framework. Moreover, add a few words of explanation about the difference among AUC and AUCpr.

We assessed the accuracy of the probability from logistic regression and PE-SCORE in the validation database using receiver operating characteristic curve and diagnostic indexes from contingency table analysis. We used precision recall and F1 index to provide additional information given the imbalance in our dataset for the outcomes of interest.

We also added this important reference: Saito, Takaya, and Rehmsmeier. The precision-recall plot is more informative than the ROC plot when evaluating binary classifiers on imbalanced datasets. *PloS One* 2015; 10(3): e0118432.

− line 283: please, add a reference for the Spiegelhalter z-test.

We added the following three references:

1) Spiegelhalter DJ. Probabilistic prediction in patient management and clinical trials. *Statistics in Medicine* 1986; 5(5): 421–33.
2) Van Calster B, McLernon DJ, van Smeden M. et al. Calibration: the Achilles heel of predictive analytics. *BMC Med* 2019; 17, 230. https://doi.org/10.1186/s12916-019-t466-7
3) Walsh CG, Sharman K, Hripcsak G. Beyond discrimination: A comparison of calibration methods and clinical usefulness of predictive models of readmission risk. *J Biomed Inform*. 2017; 76:9-18. doi:10.1016/j.jbi.2017.10.008

The section “Model development” (line 320) discusses the topic in slightly less detail than Figure 1 shows, in my opinion. For instance, in the text the part related to “other reasons of hospitalization” and “clotting disorder” is not discussed. Please, check the completeness of this section and add a few details about variable selection.

After realizing the clinical site itself was an important influence on the probability of outcomes, we looked for reasons for differences between clinical sites and determined there were significant differences between sites in the proportion of those considered to have medical or social reasons for hospitalization. In contrast, although clotting disorder was statistically significant, it was uncommon. Eventually, the clinical research site was considered as having a random effect in the final logistic model equation. We have clarified this information in the revised manuscript.

Other specific comments:

− lines 351-352 and Table 3: in the text it is stated that relative risks of the final nine variables were similar in development and validation cohorts as shown in Table 3. However, Table 3 shows odds ratios for the development cohort and relative risks for the two cohorts. Please, clarify this point.

Thank you for pointing this out. This was an error/misstatement on our part. We used multivariable logistic regression analysis to derive the final 9-variable logistic regression (LR) model. We reported on each variable in the final LR model as an odds ratio on the development database. The LR prognostic model is a formula that may be difficult for clinical providers to use. Therefore, we converted the 9 variables in the LR model into a 9-variable points based scoring model (PE-SCORE). We report on each of the 9 variables in PE-SCORE on the development and the validation databases as relative risks.

− line 352: please, clarify why 2 points are assigned to Severe Renal Impairment and 1 point is assigned to any other variable. More specifically, as at line 265 the authors stated “Points assigned were proportional to the odds ratio” and later on (lines 269-271) “we assigned whole points and weights to the final variables of the nine-point tool, which were proportional to each variable’s odds ratio for the primary outcome.” Try also to explain how points have been assigned according to that statements.

The odds ratios of most of the nine predictor variables were similar and each was assigned 1 point, except for the creatinine > 2.0 mg/dL variable, which was assigned 2 points. The reason 2 points were awarded for creatinine > 2.0mg/dL was based on the adjusted odds ratio of 5.37 for this variable. The adjusted odds ratio of 5.37 for renal impairment is more than double that of 5 variables in the model. Compared to dysrhythmia, which had the second highest adjusted odds of 4.00, the adjusted odds for creatinine elevation is 40% higher. We recognized that by awarding 2 points for creatinine elevation, the range for our point system would be 0–10, which is standard for many similar scales. The weights assigned to each variable in the final PE-SCORE model are listed in Table 3. The lowest PE-SCORE is 0 and the highest score is 10.

One of our goals was to construct a points system that was as simple as possible to be used in real time in the ED much like the sPESI and PERC rules. With our scoring system, a single sheet of paper can list the 9 variables and whether each variable is present or not (yes or no). If severe renal impairment is present, 2 points are awarded. For the other 8 variables, 1 point is awarded if the condition is present.

If any ED physicians want to use a finer scale, we have supplied the coefficients derived from the logistic regression. A computer program would be required to calculate the probability of a positive primary outcome. Also, additional data entry would be required by ED personnel. The following supplemental figure is now provided to explain how PE-SCORE would be used:

Finally, where are the weights mentioned and how are they used?

Thank you for pointing this out. We discussed points being assigned, but did not discuss the term “weights.’ We assigned more points to the variable with the highest odds ratio. We decided to assign points based on the strength of the odds ratio and prevalence.

Using the response to each of the 9 binary variables, the probability from the regression was calculated using the logistic regression (LR) equation. At the same time, we calculated the sum of the positive points for the PE-SCORE. Table 4 shows the predicted events determined by LR correspond to each total PE-SCORE, the actual number of events in the validation database at each PE-SCORE, and the proportion of patients with the primary outcome at each PE-SCORE in the development and validation databases.

We determined diagnostic accuracy of the logistic regression and PE-SCORE by assessing the receiver operating characteristic curve and precision recall using the contingency table. We compared the prognostic performance (expressed as AUC) of the LR and PE-SCORE models using De Long’s test.

− lines 351-352: again, did you mean that the 9-variables model had also been estimated on the validation cohort? That is, the variables have been kept fixed and regression coeffiecients re- estimated? If I understand correctly, can you confirm that this was done only as a support in deciding the weights of the point model? In this case, however, it seems to me that we are somehow evaluating how the model works in the validation cohort. In other words, it seems a way to also tune the points model on the validation cohort. I'm not sure I understand correctly.

We tested the 9-variable points model called PE-SCORE on the development and validation databases.

− lines 358-359: why were 95% of the development cohort and 92% of the validation cohort considered? Were these subgroups chosen at random? Lines 402-404 clarify that these are the percentages of subjects with complete records on the variable of interest: I suggest to anticipate here this information.

We tested the PE-SCORE on patients that had all components of the prognostic model available for full scoring and for complete analysis of its prognostic performance. All 9 components of the prognostic model were available for 95% of the development database and 92% of the validation database.

− line 361 and related Table 4: maybe, an instance of how to compute these values can be provided, for the sake of reproducibility. Either the methods section or the results section should give readers enough information so that they can understand and repeat these analyses. Please, always consider this rule both when reviewing the methods section and the results section.

Thank you for pointing this out. We will include the following explanation:

One point is assigned to most variables in the 9-variable PE-SCORE model if the condition is present, but 0 point is assigned if the condition is absent. The exception is creatinine > 2.0 mg/dL variable. This variable is assigned 2 points if creatinine > 2.0 mg/dL is present and 0 points if absent. In situations where one of the variables is missing (examples: PE diagnosed by VQ scan instead of by CT and CT RV:LV ratio is unavailable, or GDE unavailable (not performed or if performed, technically challenging or poor image quality prevents interpretation), there will be a modified PE-SCORE score. A modified score above 0 is already outside low-risk, whereas a modified score of 0 includes a caveat that the missing variable limits low risk stratification.

− lines 363-367: this kind of results could be better presented by a barchart like the following where the red part represents the percentage of primary events and the green part its complement. This is only a suggestion, please feel free to ignore/modify the idea. However, this representation (or any similar one you prefer) has the advantage of immediately showing the relationship between the score and the observed primary events.

We followed your suggestion and truly appreciate the generous effort to create the example. We now have 1) stacked column graphs for proportions with primary composite outcome, and 2) bar column graphs distinguishing numbers with primary composite outcome vs death at each PE-SCORE calculation for the development and validation databases. The data depicted in the figure reflects those with complete PE-SCORE scores (all 9 variables available for scoring). We reported on outcomes for those with an incomplete PE-SCORE (missing one or more variables) in the text (*Results section: prognostic model performance subsection*).

Legend for Figure:

Panels A and B show 2D stacked column charts stratified by proportions of patients with primary composite outcome positive (lower column) and negative groups (upper column) for each PE-SCORE calculation in the development and validation databases. Panels C and D show column charts for the number of patients with primary outcome positive next to the number with death for each PE-SCORE calculation in the development and validation databases.

***It is important to disclose that while creating reports to inform the primary outcome versus death outcome graphs in the figure suggested by Reviewer #3, the PI discovered a data entry error. Although this was discovered (after the databases were closed for analyses for the original manuscript) while following the reviewer suggestions, we believe it is important to disclose this error. We subsequently tested the data, factoring this pertinent corrected number with subsequent analyses, which did not alter values or calculations of percentages in univariate analyses (as rounded to one decimal point) or more complex metrics like precision, recall, or F1 index (as rounded to two decimal points).***

After explaining how to use PE-SCORE, we have added the following information to the revised manuscript:

“None of the other 8 variables used to calculate the PE-SCORE were missing for this group. A modified PE-SCORE that did not include GDE was calculated with a reduced potential range of 0–9 points. Their modified scores had an actual range of 0–4. The percentages of patients experiencing the primary outcome among those with modified PE-SCORE scores of 0, 1, 2, 3, 4 were 16.7%, 16.7%, 50.0%, 50.0%, and 0%, respectively. In comparison, the percentages of patients without missing GDE who experienced the primary outcome were 2.1%, 7.3%, 18.6%, 38.0%, and 35.6% among those with a PE-SCORE of 0, 1, 2, 3 and 4, respectively. Except for a modified score of 4, these percentages were higher in each point category for the patients with GDE missing than the same score for the group not missing GDE.“

− lines 377-378: as in Table 5 several discrimination and calibration metrics are presented, the results for each should be discussed in the text. I also noted that AUC values are compared on a descriptive basis: I suggest adding the p-value of a formal test to increase the strength of the results^7^.

^7^ By using http://support.sas.com/kb/45/339.html and http://support.sas.com/kb/25/017.html for instance, to test AUC values in either independent or correlated sample, for instance. Maybe, some test is also available to assess AUCpr values and compare AUCpr values on two (in)dependent samples.

Thank you for pointing this out to us and for the great suggestions to make this report better. Below are two additions to the revised manuscript:

1. Comparison of full logistic model (AUC 0.83) vs. points model (AUC 0.78) in the development dataset: We used the method described by DeLong^1^ and outlined for SAS here: https://support.sas.com/documentation/cdl/en/statug/63962/HTML/default/viewer.htm#statug_logistic_sect066.htm. The resulting p-value is <0.01, indicating a significant difference in the 2 ROC curves.
   - Our interpretation: Both versions of the prognostic performance are in the good range, although the AUC for the full logistic regression is significantly higher (DeLong’s test had p value < 0.01). See line numbers 344–348 and 481–483 in the revised manuscript.
2. We compared ROC curves of PE-SCORE in the development dataset (AUC 0.78) vs. validation dataset (AUC 0.77). We used the chi square test presented by Gonen.^2^ The resulting p-value was 0.49, indicating no statistically significant difference in the two ROC curves.
   - Our interpretation: The prognostic performance of PE-SCORE was similar in the development and validation databases. See line numbers 485–489 in the revised manuscript.

In addition, we have added the confidence intervals of the AUC.

^1^ DeLong, E. R., D. M. DeLong, and D. L. Clarke-Pearson. 1988. “Comparing the Areas under Two or More Correlated Receiver Operating Characteristic Curves: A Nonparametric Approach.” Biometrics 44 (3): 837–45.

^2^ Gonen, M. 2007. “Analyzing Receiver Operating Characteristic Curves with SAS.” <https://dl.acm.org/doi/abs/10.5555/1554830>.

− Table 5: in the column of the Spiegelhalter z-test I suggest to write “Spiegelhalter z test and p value”: to clarify the meaning for the two values. Alternatively, only the p-value can be indicated as in the case of the Hosmer-Lemeshow test (last column), without changing the text.

Thank you. We changed the column headings in Table 5 as you advised.

− lines 396 and following: here the discussion on the risk thresholds is started, but in lines 406-407 and following the discussion of the performance of the logistic model and of the PE-SCORE is continued. I suggest clearly separating the discussion on the logistics / points model from the discussion on risk thresholds. Maybe, the discussion of the results presented in Table 5 and Figures 3-4 can be reorganized in a more homogeneous manner.

Thank you. We have adjusted per your recommendation.

− lines 409-411: can the p-value of some test be added to these qualitative evaluations? That is, can an evaluation of the statistical significance of a slope > 1 be added? In the logistic regression framework, this can be easily done^8,9^. In the absence of this assessment, it is difficult to establish whether there is a real effect or is it just a sampling issue.

^8^ Genders TS, Steyerberg EW, Alkadhi H, Leschka S, Desbiolles L, Nieman K, et al. A clinical prediction rule for the diagnosis of coronary artery disease: validation, updating, and extension. Eur Heart J. (2011) 32:1316–30. doi: 10.1093/eurheartj/ehr014

^9^ Moons, Karel GM, et al. "Risk prediction models: II. External validation, model updating, and impact assessment." Heart 98.9 (2012): 691-698.

Thank you. We added 95% confidence intervals (CIs) for each of the slope and intercept results.

For the logistic regression model on the development database: 95% CI for slope and intercept are 0.920–1.138 and -0.040–0.027, respectively.

For PE-SCORE on development database: 95% CI for slope and intercept are 0.829–1.102 and -0.031–0.047, respectively.

For PE-SCORE on validation database: 95% CI for slope and intercept are 0.867–1.146 and -0.049–0.045, respectively.

− Table 6: please add the meaning to each letter A, B, C, D.

The meaning of each letter is now found in the table’s footnote.

− lines 429-433: here the results of the Supplementary Table 3 are presented but not discussed. Please, either add a few comments or remove this part.

Thank you for pointing this out. We added the following important information to the manuscript:

“We used it for the primary composite outcome of death and pre-defined clinical deterioration outcomes within 5 days. We identified low-risk ESC criteria, which incorporated low-risk sPESI threshold and absence of RV abnormalities (using our definitions). In the development and validation databases, low-risk sPESI had sensitivity 85.2% (79.6%–89.7%) and 80.4% (74.4%–85.5%), specificity 39.0% (35.4%–42.6%) and 43.4% (39.4%–47.6%), PPV 28.7% (27.0%–30.4%) and 34.1% (32.0%–36.3%), NPV 90.1% (86.7%–92.8%) and 85.9% (82.0%–89.0%) respectively. In the development and validation databases, low-risk ESC sensitivity 99.5% (97.4%–99.9%) and 97.7% (94.6%–99.2%), specificity 10.5% (8.3%–12.9%) and 17.2% (14.2%–20.5%), PPV 24.2% (23.8%–24.7%) and 30.1% (29.2%–31.0%), NPV 98.7% (91.4%–99.8%) and 95.3% (89.3%–98.0%), respectively. **Table 6** shows that in the development and validation databases, low-risk PE-SCORE had sensitivity 98.5% (95.2%–99.6%) and 92.4% (87.5%–95.5%), specificity 20.7% (17.7%–23.9%) and 31.5% (27.6%–35.6%), PPV 26.0% (22.9%–29.3%) and 33.0% (29.1%–37.1%) and NPV 97.9% (93.6%–99.5%) and 91.9% (86.7%–95.2%), respectively. Overall, both ESC and PE-SCORE models, which involved RV assessments, outperformed low-risk SPESI when focused on the primary composite outcomes that matter to point of care decision makers.”

Minor comments

− line 27: Add the acronym “(RV)“ here instead of line 29.

This was done.

− line 32: Please, substitute here predictive with prognostic, to make immediately clear what are you interested in predicting.

We replaced ‘predictive’ with ‘prognostic’.

− line 97: here 107 “data elements” are mentioned while at line 138, 138 variables are mentioned: could you better clarify this point, please?

Thank you. We corrected the number of data elements to 138 in the manuscript.

− line 148-149: It is not clear to me what is meant by the sentence about sensitivity analysis.

We removed the term sensitivity analysis and instead reported on the outcomes for the variable with potential missingness in the final model. Twenty-one (2.2%) of the patients in the developmental database were missing GDE. Most of these missing GDE values were marked as inadequate for interpretation. Six of these 21 had a positive primary outcome. The percentages of patients experiencing the primary outcome among those with GDE showing RVD negative, positive, and missing were 14.4%, 38.4%, and 28.6%, respectively.

− line 192: I have really appreciated this subsection! I have very rarely found sample size estimates in studies like this one.

Our goal was to adhere to TRIPOD guidelines.

− line 251: as the analysis based on the random forest approach is not presented in the section Results, but only briefly discussed in the Discussion section, I suggest to avoid any mention here.

It will be removed.

− line 276 and in the following: maybe “PE-SCORE” can be simply used without to continuously mention “points model”. It seems to me that the good acronym “SCORE” is well self-explaining.

Thank you.

− line 302: Once again, it is not clear to me what is meant by the sentence about sensitivity analysis. According to what I mean by the term “Sensitivity analysis”, the comparison of frequencies in different groups is not sensitivity analysis.

We will clarify our statement and report on the missingness of GDE and associated outcomes of those with missing GDE scores.

− line 323: please add “(p > 0.25 )” at the end of the comment about cancer and heart failure. In general, add the corresponding p-values or a meaningful summary of them in the text, to facilitate the reader and directly support the statements.

Thank you. We added p value for heart failure (p = 0.285) and for ‘any cancer’ (p = 0.987).

− line 345: Section “Model specification” can be removed, in my opinion, without loss of relevant information.

We removed it.

− Table 6: Recall=A/(A+B)

Thank you. We corrected this.

− lines 478-79: please, add the references of the 5 studies mentioned here; add also the references of the few studies including images as candidate variables.

Thank you for pointing this out. We have cited the following references.

**The five original prognostic studies with unclear number of candidate variables:**

Kostrubiec M, Pruszczyk P, Bochowicz A, et al. Biomarker-based risk assessment model in acute pulmonary embolism. Eur Heart J 2005;26:2166–72

Lankeit M, Friesen D, Schafer K, et al. A simple score for rapid risk assessment of non-high-risk pulmonary embolism. Clin 2013;102:73–80.

Jo JY, Lee MY, Lee JW, et al. Leukocytes and systemic inflammatory response syndrome as prognostic factors in pulmonary embolism patients. BMC Pulm Med 2013;13:74.

Zhu L, Wang C, Yang YH, et al. [Prognostic value of right ventricular dysfunction and derivation of a prognostic model for patients with acute pulmonary thromboembolism]. Zhonghua Liu Xing Bing Xue Za Zhi 2009;30:184–8.

Bova C, Sanchez O, Prandoni P, et al. Identification of intermediate-risk patients with acute symptomatic pulmonary embolism. Eur Respir J 2014;44:694–703.

**The references for studies including images as candidate variables are:**

Zhu L, Wang C, Yang YH, et al. [Prognostic value of right ventricular dysfunction and derivation of a prognostic model for patients with acute pulmonary thromboembolism]. Zhonghua Liu Xing Bing Xue Za Zhi 2009;30:184–8.

Bova C, Sanchez O, Prandoni P, et al. Identification of intermediate-risk patients with acute symptomatic pulmonary embolism. Eur Respir J 2014;44:694–703.

Jo JY, Lee MY, Lee JW, et al. Leukocytes and systemic inflammatory response syndrome as prognostic factors in pulmonary embolism patients. BMC Pulm Med 2013;13:74.

Jiménez D, Kopecna D, Tapson V, et al. Derivation and validation of multimarker prognostication for normotensive patients with acute symptomatic pulmonary embolism. Am J Respir Crit Care Med 2014;189:718–26.

Sanchez O, Trinquart L, Caille V, et al. Prognostic factors for pulmonary embolism: the PREP study, a prospective multicenter cohort study. Am J Respir Crit Care Med 2010;181:168–73.

Wicki J, Perrier A, Perneger TV, et al. Predicting adverse outcome in patients with acute pulmonary embolism: q risk score. Thromb Haemost 2000;84:548–52. 123.

Yamaki T, Nozaki M, Sakurai H, et al. Presence of lower limb deep vein thrombosis and prognosis in patients with symptomatic pulmonary embolism: preliminary report. Eur J Vasc Endovasc Surg 2009;37:225–31.
