# Supplementary material for: Development and validation of a prognostic tool: Pulmonary embolism short-term clinical outcomes risk estimation (PE-SCORE)
Source: PLoS One. 2021 Nov 18;16(11):e0260036. doi: 10.1371/journal.pone.0260036 (PMC8601564; doi:10.1371/journal.pone.0260036)
Supplement: Attachment — Submitted filename: ITEMIZED responses for 2nd revision PLOSONE.docx [file pone.0260036.s010.docx]

October 22, 2021

Christophe Leroyer

Academic Editor

PLOS ONE

RE: PONE-D-21-19015

Development and validation of a prognostic tool: pulmonary embolism short-term clinical outcomes risk estimation (PE-SCORE)

Thank you to the previous reviewer for approval of our previous revisions and the new reviewer for the comments and suggestions. We have responded to the new reviewer’s comments. We believe the revisions have made the report clearer and better for readers. We are pleased to submit our revised manuscript. In the “track changes” version of our enclosed manuscript, red font denotes additions to the original submission and strikethrough text denotes deletions from the original. We hope this resubmission meets with full acceptance.

Thank you for considering our work.

Best regards,

Anthony J. Weekes, MD, MSc

On behalf of the PESCOR Steering Committee

RESPONSES TO REVIEWER COMMENTS

Reviewer #2: Overall, this manuscript has significantly improved since initial submission. While some of my initial concerns about the overall usefulness of this decision tool still stand, in its current iteration this manuscript provides better explanations of how the tool variables were derived and validated and provides increased clarity about the authors’ intent for how the tool is to be used. This manuscript makes a much better argument for why efforts should be made to externally validate (PE-SCORE) and evaluate its performance outside of the institutions in which it was created. The efforts put forth by the authors to address commentary and concerns in my initial review were exhaustive. At this point in time I am satisfied with the revisions to this manuscript and the authors’ responses to my questions. No further revisions are recommended at this time.

**Thank you. We made every effort to respond to each of the reviewer’s comments and to make the manuscript clearer.**

Reviewer #4: Comments to the authors :

The authors used data from a prospective registry in which 6 emergency centers in the USA participated, to propose a prognostic score validated secondarily in a second registry.

Their score is based on 9 items, and is claimed to be able to predict a population at low risk of deterioration compared to a population at higher risk of deterioration.

The desire to develop a score with a pragmatic interest (promoting safe outpatient treatment versus keeping patients who may benefit from more intensive treatment in hospital) is commendable and the authors should be warmly congratulated.

However, I have several comments which for the moment limit the acceptability of this work.

Major comments :

- Methods : the authors chose as primary endpoint an unusual composite endpoint compared to other studies(1–3). The authors would have to justify the construction of this criterion more strongly. For example if the objective is to individualize a population to make the risk of death, we could expect death and death from pulmonary embolism as the main endpoint, as was the case with the PESI score. Conversely, if the objective is to individualize a population with more risk of degradation despite well-conducted anticoagulant treatment, the authors must justify why they did not follow criteria such as the one proposed in the PEITHO trial, for example.

**Thank you. The goal of our study was to address outcomes that occur within a time frame that is likely attributable to acute PE and for which time-sensitive decisions need to be made at the point of care. The point of care in which decisions are most challenging (after confirming the PE diagnosis) is the ambulatory or emergency department setting. The outcomes/endpoints we used are more stringent than the one used by the article you cited. The endpoints of death, recurrence of VTE, and major bleeding within 30 days are less common in the era of novel anticoagulants; we provide the data on these endpoints in our tables.**

**Our primary outcomes included death, cardiac arrest, hypotension, respiratory failure, new dysrhythmia, and reperfusion intervention within 5 days of PE diagnosis. These are outcomes that concern physicians at the point of decision-making in regards to disposition and the level of subsequent monitoring as reported by Kabrhel et al.**[^1^](https://paperpile.com/c/GyAPzc/DbAu) **The emergence of pulmonary embolism response teams in the USA and other countries has focused on identification of those at risk for acute PE related adverse events (clinical deterioration, our primary outcome) and the efficacy of interventions. Previous reports have explained the rationale and used these outcomes.**[^1–7^](https://paperpile.com/c/GyAPzc/DbAu+e2gK+acH3+tAEy+jgtC+aODU+9GLT) **Similar to Vinson et al.,**[^6^](https://paperpile.com/c/GyAPzc/aODU) **in this PE-SCORE report, “...we adopted and adapted the definitions of major 5-day inpatient adverse events from researchers at Massachusetts General Hospital.**[^1,2,8^](https://paperpile.com/c/GyAPzc/e2gK+lbG9+DbAu) **These included clot extraction or lysis (local or systemic), respiratory support (a non-rebreather mask or more), new cardiac dysrhythmia requiring treatment, use of intravenous vasopressors or inotropics, use of defibrillation or cardiopulmonary resuscitation, recurrent PE, major hemorrhage (defined below), and death.”**

**In the PEITHO study,**[^7^](https://paperpile.com/c/GyAPzc/9GLT) **the primary outcome was death or hemodynamic decompensation (or collapse) within 7 days after randomization. In the PEITHO appendix, “hemodynamic decompensation (or collapse) was defined as:**

- **need for cardiopulmonary resuscitation; or**
- **systolic blood pressure < 90 mm Hg for at least 15 minutes, or**
- **drop of systolic blood pressure by at least 40 mm Hg for at least 15 minutes with signs of end-organ hypoperfusion (cold extremities or low urinary output < 30 mL/h or mental confusion); or**
- **need for catecholamine administration to maintain adequate organ perfusion and a systolic blood pressure of > 90 mm Hg (including dopamine at the rate of > 5 micrograms/kg per minute).”**

- Population: It is surprising to find so few elderly people in the population included in the study, even though the incidence of pulmonary embolism rises after 75 years and that advanced age is a major prognostic factor(4). Do you have a reason for this? Is it explained by a selection bias?

**We do not believe there was selection bias; our study design makes selection bias unlikely. Although we did not achieve consecutive enrollments (except at the central site), enrollments occurred soon after notification of pulmonary embolism diagnosis with early screening for study inclusion and exclusion criteria. Enrollment occurred within several hours of diagnosis and without awareness of subsequent outcomes. Our cohort characteristics represent the patient population that presented to the participating emergency departments. The mean age and standard deviation of the development and validation databases were 60.3 (16.5) and 58.5 (16.7) years, respectively. At the request of the previous academic editor, we presented the proportion of patients with age > 80, which accounted for 9.8% and 8.6% of the development and validation databases, respectively.**

**The original PESI study reported those aged > 65 years accounted for 52%–59% of the development and validation cohorts.**[^9^](https://paperpile.com/c/GyAPzc/cIXK) **The proportion of patients considered advanced age (age > 65 years) in both our databases combined (including all 6 geographically separate emergency departments) was 39.3%.**

**As detailed in the meta-analysis by Elias et al., few original prognostic models developed have vetted more than 30 candidate variables. Out of 17 original prognostic models reviewed by Elias et al., only 6 models included age in the final prognostic model.**[^10^](https://paperpile.com/c/GyAPzc/8gW0)

- Results : Many studies find renal failure as a factor of poor prognosis(5), in the short term(6). The authors must be able to discuss this strongly, in particular on the possibility that their high creatinine leaves may cause loss of sensitivity.

# **Thank you for that suggestion. We added citations that support acute renal injury as a prognostic factor.**[^11,12^](https://paperpile.com/c/GyAPzc/iEhX+jT1j) **Acute renal injury was not included as a candidate variable in PESI/sPESI. In our study, we did not attempt to differentiate renal injury from renal failure. We did not use glomerular filtration rate to assess renal function and we used a modest cut-off for creatinine level evaluation for provider use. It is indeed possible a different cut-off value of creatinine or a different renal function parameter of renal function assessment may offer an optimal prognostic performance. We added such statements to the Discussion section.**

- Discussion : I think that the authors should explain more strongly how their score would make it possible to bring things not provided by the multitude of scores already proposed and those included in the international recommendations. Even if this is discussed by the authors, it is annoying not to find the advanced age and cancer, which are well known factors associated with a poor prognosis, as factors integrated in the model.

**We added the following statements (with citations) to the Discussion section:**

*“We used prospective registry databases and developed and validated an original prognostic model from a field of 138 candidate variables. The registry involved contemporaneous and early assessments for PE provoked RV abnormalities with predefined laboratory and imaging assessments, and focused on outcomes of interest to providers at the point of decision-making and to pulmonary embolism response teams.”*

*“Original clinical scores or guidelines, which were developed for outcomes of death, recurrent VTE or major bleeding 30 days or later, tend to be pragmatically modified or adapted to consider other social/medical conditions or laboratory or imaging findings instead of being used in isolation during clinical practice.*[*^4,6,13,14^*](https://paperpile.com/c/GyAPzc/nyoHL+aODU+tAEy+WcwdU) *With PE-SCORE, variables for provider discretion on social or medical reasons for hospitalization for increased monitoring and RV imaging assessment are built in.”*

**The PESI in 2005 was developed after vetting 29 candidate variables, including demographics, comorbid conditions, physical examination findings, laboratory (not including cardiac biomarkers or creatinine), and chest x-ray findings. As detailed in the meta-analysis by Elias et al., few original prognostic models developed have vetted more than 30 candidate variables.**[^10^](https://paperpile.com/c/GyAPzc/8gW0) **Out of 17 original prognostic models reviewed by Elias et al., 6 models included age in the final prognostic model, eight included cancer, and only one model included creatinine. When biomarker and imaging choices were considered as candidate variables, different parameters were used or included in the final model.**

**Our report on PE-SCORE prognostic model development and validation offers rigorous vetting of over 130 candidate variables that were contemporaneously available and feasible at the time of clinical decision-making. Imaging and biomarker definitions were predefined. In addition, we preemptively vetted age and cancer (important variables from PESI) through both univariate analysis and penalized regression analyses, but they did not achieve statistical significance in the analyses.**

**In the original derivation and validation of PESI, cancer was present in 19% and 16% of the development and validation databases, respectively. In our PE-SCORE study, cancer was present in 24.8% of our PE patients.**

**The absence of advanced age or cancer as discrete variables in PE-SCORE does not prevent these features from being considered by provider’s discretion for social or medical reasons for hospitalization or an increased level of monitoring—an important component of PE-SCORE.**

Minor comments:

- population : one of the limits is the difficulty to extrapolate the results to inpatients diagnosed with PE, which represents about a third of PE cases. Can the authors discuss it a little bit ?

**Thank you for bringing this to our attention. We focused on emergency department patients and settings where the cadence and feasibility of testing may not be generalizable to those developing acute PE while already in the inpatient setting and who may have different risk factors or susceptibilities to PE-associated deterioration from those diagnosed in an outpatient setting.** **We added such statements to the Discussion section.**

- Globally, the draft is of interest, and well written, but some of the information may be put in appendix, in order to shorten it.

**Thank you. We had revised the manuscript in response to the helpful comments and suggestions from 3 previous peer reviewers and the first academic editor. We hope these additional responses and revisions meet with acceptance.**

REFERENCES

1.	[Kabrhel C, Sacco W, Liu S, Hariharan P. Outcomes considered most important by emergency physicians when determining disposition of patients with pulmonary embolism. Int J Emerg Med. 2010;3(4):239–64.](http://paperpile.com/b/GyAPzc/DbAu)

2.	[Hariharan P, Takayesu JK, Kabrhel C. Association between the Pulmonary Embolism Severity Index (PESI) and short-term clinical deterioration. Thromb Haemost. 2011 Apr;105(4):706–11.](http://paperpile.com/b/GyAPzc/e2gK)

3.	[Rosovsky R, Zhao K, Sista A, Rivera‐Lebron B, Kabrhel C. Pulmonary embolism response teams: Purpose, evidence for efficacy, and future research directions [Internet]. Vol. 3, Research and Practice in Thrombosis and Haemostasis. 2019. p. 315–30. Available from:](http://paperpile.com/b/GyAPzc/acH3) <http://dx.doi.org/10.1002/rth2.12216>

4.	[Kabrhel C, Rosovsky R, Baugh C, Connors J, White B, Giordano N, et al. Multicenter Implementation of a Novel Management Protocol Increases the Outpatient Treatment of Pulmonary Embolism and Deep Vein Thrombosis [Internet]. Academic Emergency Medicine. 2018. Available from:](http://paperpile.com/b/GyAPzc/tAEy) <http://dx.doi.org/10.1111/acem.13640>

5.	[Weekes AJ, Johnson AK, Troha D, Thacker G, Chanler-Berat J, Runyon M. Prognostic Value of Right Ventricular Dysfunction Markers for Serious Adverse Events in Acute Normotensive Pulmonary Embolism. J Emerg Med. 2017 Feb;52(2):137–50.](http://paperpile.com/b/GyAPzc/jgtC)

6.	[Vinson DR, Drenten CE, Huang J, Morley JE, Anderson ML, Reed ME, et al. Impact of relative contraindications to home management in emergency department patients with low-risk pulmonary embolism. Ann Am Thorac Soc. 2015 May;12(5):666–73.](http://paperpile.com/b/GyAPzc/aODU)

7.	[Meyer G, Vicaut E, Danays T, Agnelli G, Becattini C, Beyer-Westendorf J, et al. Fibrinolysis for patients with intermediate-risk pulmonary embolism. N Engl J Med. 2014;370(15):1402–11.](http://paperpile.com/b/GyAPzc/9GLT)

8.	[Kabrhel C, Okechukwu I, Hariharan P, Takayesu JK, MacMahon P, Haddad F, et al. Factors associated with clinical deterioration shortly after PE. Thorax. 2014 Sep;69(9):835–42.](http://paperpile.com/b/GyAPzc/lbG9)

9.	[Aujesky D, Obrosky DS, Stone RA, Auble TE, Perrier A, Cornuz J, et al. Derivation and validation of a prognostic model for pulmonary embolism. Am J Respir Crit Care Med. 2005;172(8):1041–6.](http://paperpile.com/b/GyAPzc/cIXK)

10.	[Elias A, Mallett S, Daoud-Elias M, Poggi J-N, Clarke M. Prognostic models in acute pulmonary embolism: a systematic review and meta-analysis. BMJ Open. 2016 Apr 29;6(4):e010324.](http://paperpile.com/b/GyAPzc/8gW0)

11.	[Murgier M, Bertoletti L, Darmon M, Zeni F, Valle R, Del Toro J, et al. Frequency and prognostic impact of acute kidney injury in patients with acute pulmonary embolism. Data from the RIETE registry. Int J Cardiol. 2019 Sep 15;291:121–6.](http://paperpile.com/b/GyAPzc/iEhX)

12.	[Chopard R, Jimenez D, Serzian G, Ecarnot F, Falvo N, Kalbacher E, et al. Renal dysfunction improves risk stratification and may call for a change in the management of intermediate- and high-risk acute pulmonary embolism: results from a multicenter cohort study with external validation. Crit Care. 2021 Feb 9;25(1):57.](http://paperpile.com/b/GyAPzc/jT1j)

13.	[Roy P-M, Penaloza A, Hugli O, Klok FA, Arnoux A, Elias A, et al. Triaging acute pulmonary embolism for home treatment by Hestia or simplified PESI criteria: the HOME-PE randomized trial. Eur Heart J. 2021 Aug 31;42(33):3146–57.](http://paperpile.com/b/GyAPzc/nyoHL)

14.	[Konstantinides S, Meyer G, Becattini C. 2019 ESC Guidelines for the diagnosis and management of acute pulmonary embolism developed in collaboration with the European Respiratory Society (ERS …. Eur Heart J [Internet]. 2020; Available from:](http://paperpile.com/b/GyAPzc/WcwdU) <https://researchportal.helsinki.fi/en/publications/2019-esc-guidelines-for-the-dignosis-and-management-of-acute-pulm>
